# Prescribed opioid analgesic use in pregnancy and risk of neurodevelopmental disorders in children: A retrospective study in Sweden

**DOI:** 10.1371/journal.pmed.1004721

**Published:** 2025-09-16

**Authors:** Emma N. Cleary, Ayesha C. Sujan, Martin E. Rickert, Franziska Fischer, Tyra Lagerberg, Zheng Chang, Paul Lichtenstein, Patrick D. Quinn, Anna Sara Öberg, Brian M. D’Onofrio

**Affiliations:** 1 Department of Psychological and Brain Sciences, Indiana University, Bloomington, Indiana, United States of America; 2 Department of Anesthesiology, Perioperative and Pain Medicine, Stanford University School of Medicine, Stanford, California, United States of America; 3 Department of Medical Epidemiology and Biostatistics, Karolinska Institutet, Stockholm, Sweden; 4 Department of Psychiatry, Warneford Hospital, University of Oxford, Oxford, United Kingdom; 5 Department of Applied Health Science, School of Public Health, Indiana University, Bloomington, Indiana, United States of America; 6 Department of Epidemiology, Harvard T.H. Chan School of Public Health, Boston, Massachusetts, United States of America; London School of Hygiene and Tropical Medicine, UNITED KINGDOM OF GREAT BRITAIN AND NORTHERN IRELAND

## Abstract

**Background:**

The extent to which the documented association between prenatal prescribed opioid analgesic (POA) exposure and neurodevelopmental disorders in children is causal or due to confounding is unknown. The objective of this study was to evaluate associations between dose and duration of POA exposure during pregnancy and autism spectrum disorder (ASD) or attention-deficit/hyperactivity disorder (ADHD) in children while minimizing bias due to confounding and other sources.

**Methods and findings:**

This retrospective study analyzed a population-based cohort of births using national register data from Sweden. The ASD analysis cohort consisted of 1,267,978 children born in Sweden from July 1st, 2007 to December 31st, 2018, with follow-up through 2021. A shorter eligibility period was used to study ADHD given its later age of typical diagnosis, consisting of 918,771 children born through December 31st, 2015. Text-mining algorithms were used to derive cumulative dose and duration of POA exposure during pregnancy from filled POA prescriptions, as well as to identify prescriptions that were to be taken on an “as needed” basis. Outcomes were identified through inpatient or outpatient clinical diagnosis of ASD and ADHD or dispensed ADHD medications. Cox proportional hazards regression models were adjusted for measured covariates from multiple domains. Several designs were used to help address unmeasured confounding: comparisons with children whose birthing parent had a diagnosed painful condition but did not receive POAs, children whose birthing parent received POAs in the year before but not during pregnancy, and siblings who were not exposed to POAs. Of the 1,267,978 children, 48.6% were female and 4.4% were exposed to POAs during pregnancy. At age 10, cumulative incidence of ASD was 2.0% among children unexposed to POAs, 2.9% among children exposed to a low dose across pregnancy, and 3.6% among children exposed to a high dose. In unadjusted models (e.g., hazard ratio [HR]_high_, 1.74, 95% confidence interval [CI], 1.63, 1.87) and when accounting for measured covariates, cumulative maximum dose was associated with increased risk of ASD (e.g., HR_high_, 1.34, 95% CI, 1.24, 1.44). However, the associations were largely or fully attenuated when using alternative designs (particularly when comparing to children whose birthing parent received POAs before but not during pregnancy: HR_high_, 1.10, 95% CI, 1.00, 1.21). No associations were observed in the sibling comparison (HR_high_, 0.99, 95% CI, 0.81, 1.21). This overall pattern of associations was also observed when considering duration of exposure, and in numerous sensitivity analyses, as well as for analyses of ADHD. A main limitation of this study was that the distribution of dose and duration of POAs prescribed to birthing parents in Sweden limited our ability to explore the effects of extremely high dose and duration on risk for neurodevelopmental disorders.

**Conclusions:**

While increased risks with high amounts of POA exposure cannot be ruled out, the results suggest that confounding may largely explain the increased risks of ASD and ADHD associated with prenatal POA exposure at the levels observed in this cohort.

## Author summary

**Why was this study done?:**

- Prescribed opioid analgesics (POAs) are commonly prescribed to help manage pain that is experienced during pregnancy
- It is unclear whether POA exposure during pregnancy increases risk for neurodevelopmental disorders
- We examined the extent to which POA use during pregnancy increases risk for two common neurodevelopmental disorders, autism spectrum disorder (ASD) and attention-deficit/hyperactivity disorder (ADHD)

**What did the researchers do and find?:**

- We analyzed a large cohort of births in Sweden and used multiple methods to account for differences in background characteristics between children that were exposed and unexposed to POAs during pregnancy
- Our results indicated that the absolute risks of ASD and ADHD were relatively low, but that risk was greater with greater cumulative dose of exposure
- When accounting for differences in background characteristics, we found that the associations between POA exposure and ASD and ADHD were substantially weakened or no longer present

**What do these findings mean?:**

- Because most children were only exposed to one POA prescription during pregnancy, we were not able to explore the impacts of extremely high dose and duration of exposure, however, we found little evidence that POA exposure during pregnancy at the levels observed in our cohort substantially increased risk for neurodevelopmental disorders

## Abstract 3

In a retrospective analysis of national register data from more than 1.2 million children born in Sweden, Emma Cleary and colleagues investigated whether exposure to prescribed opioid analgesics during pregnancy associated with autism spectrum disorder or attention-deficit/hyperactivity disorder in offspring.

Pain affects many birthing parents and, left untreated, can have a substantial negative impact on their daily activities and quality of life [1–3]. Treatment with prescribed opioid analgesics (POAs) has decreased in recent years [4] but remains common during pregnancy. Estimates of the prevalence of POA exposure range from 3% in Norway from 2005 through 2015 [5] to 9% among pregnant Medicaid beneficiaries in the United States as of 2019 [6]. Patients and prescribers are faced with difficult decisions when weighing the risks and benefits of POA use in pregnancy. Studies of rodents indicate that prenatal opioid exposure affects cellular and molecular processes during critical periods of central nervous system development, leading to structural and functional alterations that may underlie increased risk for two of the most common neurodevelopmental disorders, autism spectrum disorder (ASD) and attention-deficit/hyperactivity disorder (ADHD) [7,8]. Several studies in humans have observed elevated risks for neurodevelopmental disorders in children after prenatal POA exposure, particularly for higher doses and long periods of exposure [9,10], but more research is needed to determine if these associations are due to causal effects of the medication or confounding, as well as to examine other sources of bias.

Few studies have examined associations between prenatal POA exposure and the specific neurodevelopmental disorders ASD [9–11] and ADHD [9,10,12], and the overall conclusions have been inconsistent. For example, a nationwide birth cohort study in South Korea observed a 7% excess risk of neuropsychiatric disorders broadly, which was entirely attenuated in a sibling comparison [10]. A retrospective study of commercial health insurance data in the United States observed elevated risks of neurodevelopmental disorders in children exposed for longer duration (≥14 days associated with 70% excess risk) and at higher doses (≥37.5 morphine milligram equivalents associated with 22% excess risk) during pregnancy [9]. Similarly, a study using a prospective, population-based pregnancy cohort in Norway found a comparable increased risk (60%) for ADHD with long duration of exposure (≥5 weeks) [12].

Inference from previous studies is limited by several factors. First, measurement error of POA exposure may have biased results. Studies that rely on self-report measures of POA use [12] may be subject to bias from birthing parents’ underreporting their prescription opioid use during pregnancy [13,14]. Studies relying on prescription databases frequently do not capture information on “as needed” dosing or other instructions that may affect the way that birthing parents take their medication. Second, previous studies have varied in how they defined their outcomes. While some studies focused solely on risk for individual disorders like ADHD [12], others combined a broad range of heterogeneous outcomes [9,10], limiting understanding of the specific risks for ASD and ADHD. Third, while all studies included measured covariates to control confounding and several further addressed confounding by indication [15] by restricting cohorts based on indications for POA use [9,12], studies varied in their ability to account for confounding from unmeasured genetic and environmental factors when assessing specific diagnoses [16,17]. Only one study employed a sibling comparison, but this analysis combined neurodevelopmental and other psychiatric conditions [10]. Finally, there are concerns related to generalizability of the existing studies. For example, selection bias and loss to follow-up may affect findings in prospective cohort studies [12].

The primary aim of this study was to examine the impact of POA use during pregnancy on risk for ASD and ADHD in children independent of confounding. This study helps to address critical gaps in the extant literature [18]. First, we supplemented covariate adjustment for a range of measured confounding factors with multiple designs to help address unmeasured confounding. We used alternative comparison groups, including children born to birthing parents with diagnosed painful conditions without POAs, children with a birthing parent with POA prescriptions in the year before pregnancy but not during pregnancy, and unexposed siblings [19]. Second, we used text-mining of prescription data to derive multiple indexes of dose and duration of exposure, as well as distinguish prescriptions for use “as needed” compared to prescriptions for consistent use. Third, we indexed ASD and ADHD using validated clinical diagnoses and ensured all included in the study had adequate follow-up time. Finally, we estimated associations between prenatal POA exposure and ASD and ADHD in children using a large, national cohort with minimal loss to follow-up.

## Methods

### Sample

In this cohort study, we analyzed data from all births in Sweden between July 1st, 2007, and December 31st, 2018, to examine the associations between prenatal POA exposure and the risk of ASD, with follow-up extending through December 31st, 2021. The age range of follow-up was from 3 years to 14.5 years (see S1 Table for sequential exclusions). For ADHD, we analyzed data from all births in Sweden from July 1st, 2007, to December 31st, 2015, again with follow-up through December 31st, 2021. This allowed for adequate follow-up time (at least 6 years) given that ADHD is typically diagnosed later in childhood than ASD [20,21]. We obtained data by linking information from Swedish population registers (S2 Table) [22–27]. This study is reported as per the Strengthening the Reporting of Observational Studies in Epidemiology (STROBE) guideline (see S3 Table). A description of our planned analyses and changes made during peer review is included in the supporting information (S1 Text).

### Exposure

We identified filled POA prescriptions using Anatomical Therapeutic Chemical (ATC) codes starting with “N02A” (see S4 Table) that were dispensed within the year before conception through the day before birth. To obtain more precise measures of POA exposure, we used natural language processing of free-text prescription information written by prescribers (detailed information on text-mining in S2 Text). The free-text information frequently contained dosage ranges (such as 1−2 tablets per day) and frequency ranges (2−4 times daily). Expanding upon an approach previously applied to ADHD medications [28], we obtained the following predicted variables: the prescribed minimum and maximum daily dosage, if the medication was prescribed on an “as needed” basis, and the duration of the prescription based on the number of pills dispensed and assuming the individual either used the maximum or minimum dose. Internal and external validation of the algorithm results using a randomly selected sample of prescriptions suggested that overall accuracy was excellent for each of the predicted variables (correlations 0.95–0.99) between the text-mining models and information extracted by independent manual raters.

To standardize the measurement of opioid exposure across different types, dosages, and formulations of opioids, each prescription was converted to oral morphine equivalent (OME) dosage (S2 Text) [29]. Prescriptions dispensed during overlapping dates were assumed to be taken concurrently, except for overlapping prescriptions with the same ATC, strength, and formulation (both immediate or both extended release), for which the start date of the later prescription was shifted so treatment intervals did not overlap—that is, we assumed that “stockpiling” of the medication occurred (S1 and S2 Figs). Our main exposure variable was the predicted maximum cumulative oral morphine milligram equivalent (referred to as cumulative dose) across the pregnancy, defined as conception date through the day before birth. We truncated the variable by excluding the top 0.1% of cumulative OME values to address potential bias from extreme values (see S1 Table). We used a categorical measure (unexposed, low dose, high dose) based on the median value (162 mg). For the analysis focused on duration, we calculated the total duration based on the predicted maximum use of the filled prescription during the pregnancy period, categorized into 1–7, 8–14, and 15+ day bins.

### Outcomes

We evaluated associations between POA exposure and neurodevelopmental disorders, identified through child ADHD medication prescriptions [23,24] and validated clinical (International Classification of Diseases [ICD]-10-based) diagnosis of ASD and ADHD (S5 and S6 Tables) [26,30,31]. Although children may have received diagnoses earlier, in the primary analysis, we only considered the first ASD diagnosis made after age two [32] and the first ADHD diagnosis made after age four [33] to improve confidence in diagnosis validity and stability.

### Covariates

We included covariates associated with POA use during pregnancy and neurodevelopmental disorders, consistent with previous research [34,35]. We included covariates for birth order, year of birth, multiple birth, sex, and birthing parent country of origin. Swedish national register data do not include information on race/ethnicity. To control for effects of other potentially harmful exposures during pregnancy we also included birthing parent smoking before and during pregnancy and exposure to other psychoactive medications in the year before conception and during pregnancy (S5 Table). We also included birthing and non-birthing parent characteristics: age at child’s birth, psychiatric diagnoses before conception (S6 Table), highest level of education before conception, cohabitation status at childbirth, and birthing parent income before conception.

### Painful conditions

We identified indications for POA treatment through specialist diagnoses of birthing parent painful conditions [36] in the year before conception and during pregnancy. These included musculoskeletal disorders, pregnancy-related conditions, injuries, and others (S7 Table).

### Statistical analyses

#### Primary analyses.

Children were followed from age two for ASD and four for ADHD until whichever came first for each respective diagnosis: first recorded emigration from Sweden, death, or end of study. To account for this censoring, we used survival analysis to evaluate associations between POA exposure and first outcome diagnosis, with child age as the timescale. We first used the Kaplan–Meier (KM) method to estimate the cumulative incidence of ASD and ADHD. We then estimated hazard ratios (HRs) by fitting Cox proportional hazards regressions models for each outcome, using robust standard errors to account for clustering of individuals with the same birthing parent. We visually inspected Schoenfeld residuals and did not detect any strong violations of the proportional hazards assumption [37].

We fit five models to estimate associations between POA exposure and each outcome. The first two models used the population-based cohorts, with unexposed births as the comparison group. In Model 1, unadjusted associations were estimated for each level of dose and duration. Model 2 added all measured covariates. We used multiple imputation to replace missing covariate data (<7%). Five full-data imputations were generated using the Markov Chain Monte Carlo procedure of SAS, version 9.4 (SAS Institute Inc).

Models 3–5 used alternative comparison groups, supplemented with covariate adjustment. Model 3 compared exposed children born to birthing parents with diagnosed painful conditions to unexposed children born to birthing parents with diagnosed painful conditions without POAs. Model 4 compared children exposed during pregnancy to unexposed children whose birthing parent had POAs in the year before conception but not during pregnancy. This model accounts for confounding by all factors that are shared by those who fill POA prescriptions around the time of pregnancy without relying on accurately capturing diagnosed conditions [38,39]. Model 5 addressed familial confounding by comparing differentially exposed siblings using a stratified Cox (i.e., fixed-effects) regression conditioned on the birthing parent [40].

#### Sensitivity analyses.

We included nine sensitivity analyses to evaluate the robustness of the assumptions underlying our primary analysis. First, we refit models 1–5 using different exposure definitions to examine whether our conclusions depended on our use of the maximum daily dose or duration in the main analyses. In sensitivity analysis 1, we examined whether the presence of “as needed” prescriptions influenced our estimates by excluding such prescriptions in our index of the predicted maximum daily dosage. In analysis 2, we examined whether the assumption of the maximum use of the filled prescriptions influenced our conclusions by basing the exposure variables on the minimum predicted daily dosage and treating the minimum value for any “as needed” prescriptions as zero.

In analysis 3, we examined whether the results from the text mining could bias our conclusions. Specifically, we repeated models 1–5 using the number of prescriptions filled during pregnancy (0, 1, or 2+), which did not rely on the algorithm’s interpretation of textual information.

We also included two other exposure definitions. In analysis 4, we defined exposure based on the first two trimesters of pregnancy only. This addressed the non-fixed length of the third trimester and removed a period of time where there may not be an opportunity for exposure should POA exposure increase risk for preterm birth [41]. In analysis 5, we examined whether our handling of overlapping prescriptions influenced the results by disregarding stockpiling—that is, we did not extend the treatment length of a prescription by the amount it overlapped with the treatment length of a previous, similar prescription but instead ignored that overlap (see S2 Text). In analysis 6, we examined dose and duration of exposure continuously.

Results of a previous study indicated increased risk for neuropsychiatric disorders following POA exposure in early pregnancy [10]. In analysis 7, we tested the hypothesis that early (first trimester) exposure to POAs would be associated with increased risk for ASD and ADHD compared to exposure in middle/late gestation (see conception date and trimester definitions in S3 Text). Specifically, we fit Model 3 and Model 4 with separate indexes for POA use in the first and second/third trimesters, comparing the statistical significance of the difference between the estimates using a Wald test.

In the primary analysis, we included covariates capturing co-medication use and smoking during pregnancy to help account for confounding factors. In analysis 8, we used a more conservative approach including covariates measured before conception only to ensure that the potential confounding factors occurred before the exposure.

Finally, in analysis 9, we repeated the main analyses with follow-up starting at birth. The earliest recorded diagnosis of ASD and ADHD was considered the incident event. This sensitivity analysis addressed potential bias introduced from consideration ASD and ADHD diagnoses after age two and four, respectively, in the primary analysis.

All data were analyzed using SAS, version 9.4 (SAS Institute). This study was approved by the Indiana University Institutional Review Board and the Swedish Ethical Review Authority. The study used data available from national registers, and informed consent was not necessary.

## Results

Among 1,267,978 children (98.5% of the target population of all children born in Sweden between July 2007 and December 2018), 616,080 (48.6%) were female and 55,427 (4.4%) were exposed to POAs during pregnancy. Among birthing parents of exposed children, 72.3% filled only 1 POA prescription during pregnancy (see S8 and S9 Tables). For sample sizes and exposure distributions across models, see S10 Table). The maximum cumulative dose ranged from 0.53 to 28,040 mg (interquartile range [IQR]: 72–312 mg, median value: 162 mg). The duration based on the maximum dose ranged from 1 to 288 days (IQR: 5–25 days, median value: 10 days). Compared with birthing parents of unexposed children, birthing parents of exposed children were more likely to smoke during pregnancy (10.5% versus 5.2%) and use a variety of psychoactive medications during pregnancy (e.g., 2.9% versus 0.4% used benzodiazepine derivatives). They were also more likely to have a psychiatric diagnosis before conception (e.g., 16.0% versus 6.9% were diagnosed with an anxiety disorder; see Table 1 for birthing parent and cohort characteristics, S11 Table for non-birthing parent characteristics, and S12 Table for background characteristics for dose and ASD sibling comparison).

**Table 1:** Key cohort characteristics.

	POA Unexposed		POA Exposed	
	*N*	%	*N*	%
	1,212,551	95.6	55,427	4.4
**Pregnancy-related characteristics**				
**Birth order**				
1st	526,860	43.5	21,240	38.3
2nd	448,315	37.0	19,573	35.3
3rd	165,017	13.6	9,392	16.9
4th or higher	72,359	6.0	5,222	9.4
**Year of birth**				
2007–2010	357,520	29.5	17,024	30.7
2011–2014	419,384	34.6	19,702	35.6
2015–2018	435,647	35.9	18,701	33.7
**Multiple births**	32,965	2.7	2,268	4.1
**Female**	589,129	48.6	26,951	48.6
**Birthing parent smoking in 3 months before pregnancy**				
None	994,583	82.0	40,770	73.6
1–9 cigarettes per day	82,373	6.8	5,302	9.6
10 or more cigarettes per day	76,780	6.3	6,679	12.1
Missing	58,815	4.9	2,676	4.8
**Birthing parent smoking in 1st trimester**				
None	1,090,993	90.0	46,963	84.7
1–9 cigarettes per day	49,344	4.1	4,300	7.8
10 or more cigarettes per day	12,892	1.1	1,499	2.7
Missing	59,322	4.9	2,665	4.8
**Exposure to other psychoactive medications**				
ADHD medication before	5,347	0.4	806	1.5
ADHD medication during	2,028	0.2	383	0.7
Anticonvulsants before	9,775	0.8	2,071	3.7
Anticonvulsants during	5,493	0.5	943	1.7
Lithium before	1,046	0.1	138	0.3
Lithium during	628	0.1	79	0.1
Antipsychotics excluding lithium before	5,425	0.5	820	1.5
Antipsychotics excluding lithium during	3,324	0.3	442	0.8
Non-benzodiazepine anxiolytics before	25,818	2.1	3,267	5.9
Non-benzodiazepine anxiolytics during	3,793	0.3	712	1.3
Benzodiazepine derivatives before	16,069	1.3	3,242	5.9
Benzodiazepine derivatives during	4,231	0.4	1,628	2.9
Benzodiazepine-related agents (z-drugs) before	23,924	2.0	4,785	8.6
Benzodiazepine-related agents (z-drugs) during	6,160	0.5	2,446	4.4
Cyclic antidepressants before	4,637	0.4	1,623	2.9
Cyclic antidepressants during	1,023	0.1	487	0.9
Non-benzodiazepine hypnotics/sedatives before	14,594	1.2	2,547	4.6
Non-benzodiazepine hypnotics/sedatives during	9,118	0.8	2,374	4.3
Migraine medications before	16,534	1.4	3,873	7.0
Migraine medications during	6,900	0.6	2,609	4.7
Medications for nicotine/alcohol use disorder before	2,302	0.2	362	0.7
Medications for nicotine/alcohol use disorder during	324	0.0	75	0.1
Other pain medications before	2,610	0.2	673	1.2
Other pain medications during	920	0.1	247	0.5
SSRIs before	74,244	6.1	8,446	15.2
SSRIs during	41,264	3.4	5,057	9.1
Paracetamol before	60,810	5.0	10,564	19.1
Paracetamol during	33,452	2.8	13,841	25.0
NSAIDs before	115,055	9.5	14,335	25.9
NSAIDs during	10,209	0.8	3,551	6.4
**Birthing parent characteristics**				
**Birthing parent age**				
19 or younger	15,581	1.3	484	0.9
20–29	516,361	42.6	23,315	42.1
30–39	631,006	52.0	28,903	52.2
40–45	48,066	4.0	2,648	4.8
46 and older	1,537	0.1	77	0.1
**Birthing parent diagnoses before conception**				
Attention-deficit/hyperactivity disorder	11,303	0.9	1,407	2.5
Autism spectrum disorder	2,420	0.2	242	0.4
Definite or uncertain suicide attempt	31,535	2.6	3,662	6.6
Alcohol use disorder	26,826	2.2	2,441	4.4
Other non-tobacco substance use disorder	10,508	0.9	1,447	2.6
Serious mental illness	9,587	0.8	1,153	2.1
Non-bipolar mood disorder	58,358	4.8	6,655	12.0
Anxiety disorder	83,774	6.9	8,856	16.0
**Highest level of education**				
Less than 9 years	32,883	2.7	1,445	2.6
9 years	92,531	7.6	6,989	12.6
1–3 years of upper secondary	419,412	34.6	23,792	42.9
Any post-secondary or postgraduate	589,218	48.6	21,271	38.4
Missing	78,507	6.5	1,930	3.5
**Any pain conditions** * not a covariate	437,032	36.0	40,972	73.9
**Country of origin is Sweden**	900,228	74.2	43,627	78.7
**Other familial and socioeconomic characteristics**				
**Cohabitation at childbirth**				
Cohabitating	1,083,465	89.4	48,628	87.7
Single	22,533	1.9	1,520	2.7
Other cohabitation situation	51,489	4.3	2,746	5.0
Missing	55,064	4.5	2,533	4.6
**Birthing parent income in year before conception**				
1st quintile (lowest)	222,040	18.3	9,103	16.4
2nd quintile	389,073	32.1	20,167	36.4
3rd quintile	290,056	23.9	13,844	25.0
4th quintile	162,851	13.4	7,104	12.8
5th quintile (highest)	102,174	8.4	4,311	7.8
Missing	46,357	3.8	898	1.6

*Abbreviations:* POA, prescribed opioid analgesic; ADHD, attention-deficit/hyperactivity disorder; SSRIs, selective serotonin reuptake inhibitors; NSAIDs, nonsteroidal anti-inflammatory drugs.

### Associations with ASD

**Fig 1:**
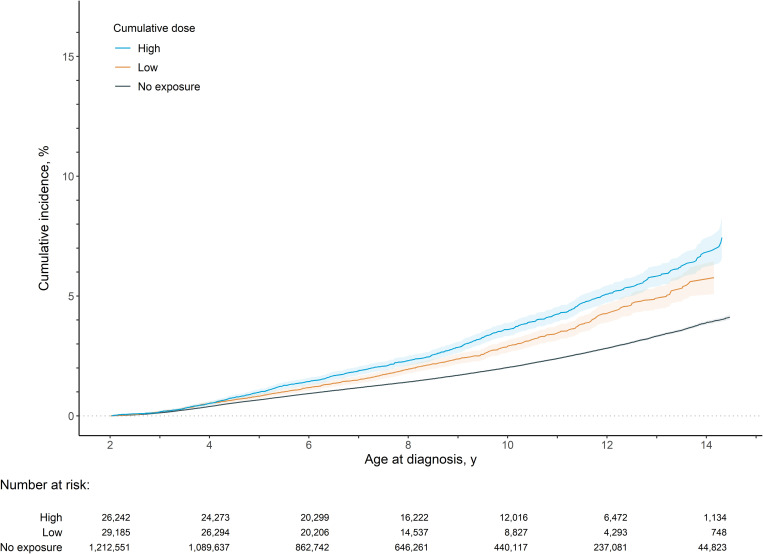
Kaplan–Meier estimates of cumulative incidence for autism spectrum disorder by cumulative dose exposure level with 95% confidence intervals.

In the ASD analysis cohort (follow-up time IQR: 5.6–11.4 years), the cumulative incidence of a diagnosis of ASD was 2.07% by the age of 10 years old. Fig 1 presents the KM estimates of ASD based on maximum cumulative dose categories. Fig 1 highlights the low absolute risk, and higher relative risk of ASD with increasing cumulative dose in the population. See S3 and S4 Figs for KM estimates for the painful conditions and before pregnancy comparison groups.

Table 2 presents the results of the five models. In model 1, cumulative maximum dose was associated with increased risk of ASD (HR_low_ = 1.42; HR_high_ = 1.74). In model 2, the associations were attenuated but POA exposure was still associated with ASD after covariate adjustment (HR_low_ = 1.21; HR_high_ = 1.34). In model 3, the comparison with children of birthing parents with painful conditions led to further attenuation (HR_low_ = 1.14; HR_high_ = 1.25). Moreover, the results of model 4, the comparison with those with before pregnancy POA exposure (HR_low_ = 0.99; HR_high_ = 1.10), and model 5, the comparison of differentially exposed siblings (HR_low_ = 1.05; HR_high_ = 0.99) resulted in estimates close to and not statistically significantly different from the null.

**Table 2:** Primary analysis of associations between POA exposure and neurodevelopmental disorders.

	HR (95% CI)				
	1. Unadjusted	2. Covariate adjusted	3. Painful conditions	4. Before pregnancy	5. Sibling comparison
**Autism spectrum disorder (ASD)**					
Dose					
Unexposed	Reference	Reference	Reference	Reference	Reference
Low	1.42 (1.31, 1.54)	1.21 (1.12, 1.31)	1.14 (1.04, 1.25)	0.99 (0.90, 1.09)	1.05 (0.87, 1.27)
High	1.74 (1.63, 1.87)	1.34 (1.24, 1.44)	1.25 (1.15, 1.36)	1.10 (1.00, 1.21)	0.99 (0.81, 1.21)
Duration					
Unexposed	Reference	Reference	Reference	Reference	Reference
1–7 days	1.42 (1.30, 1.55)	1.22 (1.11, 1.33)	1.12 (1.01, 1.24)	0.99 (0.89, 1.10)	1.06 (0.85, 1.33)
8–14 days	1.56 (1.41, 1.73)	1.33 (1.20, 1.48)	1.29 (1.15, 1.46)	1.07 (0.95, 1.21)	0.98 (0.76, 1.25)
15+ days	1.76 (1.63, 1.90)	1.29 (1.19, 1.41)	1.21 (1.10, 1.34)	1.08 (0.97, 1.20)	1.01 (0.81, 1.27)
**Attention-deficit/hyperactivity disorder (ADHD)**					
Dose					
Unexposed	Reference	Reference	Reference	Reference	Reference
Low	1.71 (1.62, 1.81)	1.35 (1.28, 1.43)	1.25 (1.17, 1.34)	1.07 (1.00, 1.14)	1.04 (0.90, 1.19)
High	1.89 (1.80, 1.98)	1.26 (1.19, 1.33)	1.21 (1.14, 1.29)	1.06 (0.99, 1.14)	0.94 (0.81, 1.09)
Duration					
Unexposed	Reference	Reference	Reference	Reference	Reference
1–7 days	1.61 (1.51, 1.72)	1.29 (1.21, 1.37)	1.21 (1.12, 1.30)	1.01 (0.94, 1.09)	1.07 (0.91, 1.27)
8–14 days	1.76 (1.64, 1.90)	1.38 (1.28, 1.49)	1.25 (1.15, 1.37)	1.10 (1.01, 1.20)	1.12 (0.92, 1.35)
15+ days	2.00 (1.89, 2.11)	1.27 (1.20, 1.35)	1.23 (1.15, 1.32)	1.09 (1.01, 1.17)	0.84 (0.71, 0.99)

*Note:* Dose and duration values are cumulative throughout pregnancy and calculated based on maximum predicted use. Models 1 and 2 are population wide. Model 3 is a subset of the population and only includes children born to those with a diagnosed painful condition. Model 4 compared children exposed during pregnancy to unexposed children whose birthing parents had POAs in the year before conception but not during pregnancy. Model 5 compares differentially exposed siblings. Models 2–5 control for all variables listed in Table 1 and non-birthing parent characteristics listed in S11 Table.

A similar pattern was observed for duration. Longer duration was associated with ASD (e.g., HR_1–7_ = 1.42; HR_8–14_ = 1.56; HR_15+ _= 1.76 in model 1), but the associations were attenuated to the null when accounting for measured covariates and using design-based confounding control (e.g., HR_1–7_ = 1.06; HR_8–14_ = 0.98; HR_15+ _= 1.01 in model 5).

### Associations with ADHD

**Fig 2:**
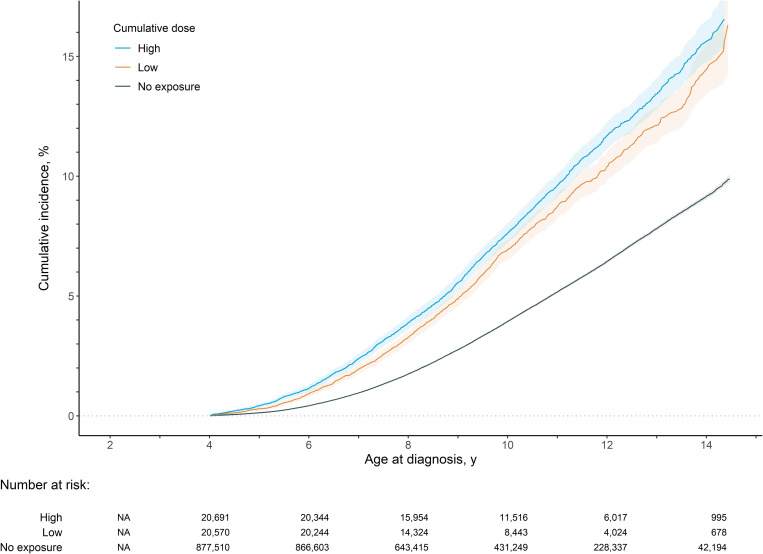
Kaplan–Meier estimates of cumulative incidence for attention-deficit/hyperactivity disorder by cumulative dose exposure level with 95% confidence intervals.

Among 918,771 children in the ADHD analysis (follow-up time IQR: 7.9–12.1 years), the cumulative incidence of diagnosed ADHD at age 10 was 4.09%. Fig 2 presents the KM estimates of ADHD based on maximum cumulative dose categories. Fig 2 illustrates a higher risk of ADHD with increasing cumulative dose. See S5 and S6 Figs for KM estimates for the painful conditions and before pregnancy comparison groups. Commensurate with the results for ASD, the model 1 results showed a dose-response association (HR_low_ = 1.71; HR_high_ = 1.89, see Table 2). As with ASD analyses, adjustment for confounding led to attenuation across the different models, and no association remained among discordant siblings (HR_low_ = 1.04; HR_high_ = 0.94 in model 5). A similar pattern of results was observed for duration.

### Sensitivity analyses

In the first set of sensitivity analyses (Tables 3 and 4 for dose and number of prescriptions and S13 Table for duration), we observed largely similar patterns to the main results when we used different indexes of POA exposure (excluding “as needed” prescriptions, based on the minimum use of POAs, and using number of filled prescriptions). Restricting exposure to the first two trimesters (S14 Table), not shifting overlapping prescriptions (S15 Table), and analyzing dose and duration continuously (S16 Table) revealed a largely similar pattern of results. There were no statistically significant differences in associations between exposure in the first trimester versus middle/late pregnancy with ASD (S17 and S18 Tables). Although most results for ADHD were not statistically significant, we observed some excess risk of ADHD with later exposure versus early exposure for high dose. No increased risks based on timing were observed for duration of exposure. Overall, these findings do not support the hypothesis that exposure to POAs early in pregnancy increases risk for ASD or ADHD. Finally, a similar pattern of attenuation was observed when adjusting for covariates measured before conception only (S19 Table) and when starting follow-up at birth using the earliest diagnosis outcome definition (S20 Table). A list of model parameters (S21 Table) and SAS version 9.4 code for the primary analyses and continuous sensitivity analyses (S4 Text) is included in the supporting information.

**Table 3:** Sensitivity analyses testing associations between dose of POA exposure and ASD.

	HR 95% CI				
	1. Unadjusted	2. Covariate adjusted	3. Painful conditions	4. Before pregnancy	5. Sibling comparison
**1:** Dose based on maximum use without including “as needed” prescriptions					
Unexposed	Reference	Reference	Reference	Reference	Reference
Low	1.39 (1.22, 1.58)	1.15 (1.01, 1.31)	1.03 (0.88, 1.20)	0.94 (0.82, 1.07)	1.05 (0.77, 1.44)
High	1.85 (1.68, 2.03)	1.35 (1.21, 1.49)	1.27 (1.13, 1.42)	1.13 (1.01, 1.26)	1.05 (0.80, 1.38)
**2:** Dose based on minimum predicted use					
Unexposed	Reference	Reference	Reference	Reference	Reference
Low	1.39 (1.24, 1.56)	1.16 (1.03, 1.30)	1.04 (0.90, 1.19)	0.94 (0.84, 1.07)	1.05 (0.79, 1.40)
High	1.93 (1.75, 2.13)	1.37 (1.23, 1.52)	1.30 (1.16, 1.47)	1.16 (1.04, 1.30)	1.08 (0.81, 1.43)
**3.** Number of prescriptions					
Unexposed	Reference	Reference	Reference	Reference	Reference
1	1.45 (1.37, 1.55)	1.26 (1.18, 1.34)	1.17 (1.09, 1.27)	1.01 (0.92, 1.10)	1.07 (0.91, 1.26)
2+	1.93 (1.77, 2.12)	1.33 (1.21, 1.47)	1.27 (1.14, 1.42)	1.14 (1.01, 1.28)	0.89 (0.68, 1.16)

*Note:* Dose and duration values are cumulative throughout pregnancy and calculated based on maximum predicted use. Models 1 and 2 are population wide. Model 3 is a subset of the population and only includes children born to those with a diagnosed painful condition. Model 4 compared children exposed during pregnancy to unexposed children whose birthing parents had POAs in the year before conception but not during pregnancy. Model 5 compares differentially exposed siblings. Models 2–5 control for all variables listed in Table 1 and non-birthing parent characteristics listed in S11 Table.

**Table 4:** Sensitivity analyses testing associations between dose of POA exposure and ADHD.

	HR 95% CI				
	1. Unadjusted	2. Covariate adjusted	3. Painful conditions	4. Before pregnancy	5. Sibling comparison
**1:** Dose based on maximum use without including “as needed” prescriptions					
Unexposed	Reference	Reference	Reference	Reference	Reference
Low	1.73 (1.59, 1.89)	1.29 (1.18, 1.41)	1.19 (1.07, 1.32)	1.03 (0.95, 1.14)	0.99 (0.79, 1.24)
High	1.94 (1.81, 2.08)	1.23 (1.14, 1.32)	1.20 (1.10, 1.30)	1.06 (0.99, 1.15)	1.01 (0.82, 1.23)
**2:** Dose based on minimum predicted use					
Unexposed	Reference	Reference	Reference	Reference	Reference
Low	1.69 (1.57, 1.83)	1.29 (1.19, 1.40)	1.19 (1.09, 1.31)	1.03 (0.95, 1.12)	1.05 (0.86, 1.29)
High	2.00 (1.87, 2.15)	1.20 (1.12, 1.30)	1.18 (1.08, 1.29)	1.07 (0.98, 1.15)	0.98 (0.79, 1.22)
**3.** Number of Prescriptions					
Unexposed	Reference	Reference	Reference	Reference	Reference
1	1.60 (1.53, 1.67)	1.28 (1.22, 1.34)	1.19 (1.13, 1.26)	1.01 (0.95, 1.07)	1.04 (0.92, 1.17)
2+	2.32 (2.18, 2.47)	1.36 (1.27, 1.46)	1.31 (1.21, 1.42)	1.19 (1.10, 1.29)	0.94 (0.77, 1.14)

*Note:* Dose and duration values are cumulative throughout pregnancy and calculated based on maximum predicted use. Models 1 and 2 are population wide. Model 3 is a subset of the population and only includes children born to those with a diagnosed painful condition. Model 4 compared children exposed during pregnancy to unexposed children whose birthing parents had POAs in the year before conception but not during pregnancy. Model 5 compares differentially exposed siblings. Models 2–5 control for all variables listed in Table 1 and non-birthing parent characteristics listed in S11 Table.

## Discussion

In this cohort study of more than 1.2 million children in Sweden, we examined the associations between multiple indexes of cumulative dose and duration of POA exposure during pregnancy and the risks of ASD and ADHD using multiple approaches to account for confounding. At the population level, we replicated previous findings [9,10,12] showing that risk of neurodevelopmental disorders increases as the dose, duration, and number of prescriptions increases. However, when we combined statistical controls for a wide range of parental and familial factors with the design comparing to children whose birthing parent received POAs before but not during pregnancy and the sibling comparison, we found that the associations between the majority of indexes of POA exposure and both ASD and ADHD were largely attenuated and did not differ statistically from the null. These are the strongest designs (models 4 and 5) as they address sources of measured and unmeasured confounding. Therefore, these results suggest that confounding may largely account for the excess risk for ASD and ADHD observed in children exposed to prenatal POAs at the levels prescribed in our cohort. These results also indicate that studies that relied solely on controlling for statistical covariates when examining dose and duration [9,10] may have overestimated the causal effects of POAs on ASD and ADHD. The results also further extend the findings of a previous study that conducted a sibling comparison to estimate risk of psychiatric and neurodevelopmental disorders broadly defined [10]. It is important to note, however, that the majority of children were exposed to only 1 prescription during pregnancy. Based on the distribution of dose and duration as prescribed to birthing parents in Sweden, we were not able to explore the effects of extremely high dose and duration. Based on the width of the confidence intervals for high dose and duration of POA exposure across our models, we cannot rule out that greater POA quantities may increase risk for neurodevelopmental disorders.

This study adds to the existing literature in several ways. First, we used validated indexes of dose and duration identified through a free-text-mining algorithm, allowing us to identify prescriptions for “as needed” use. To our knowledge, this is the first study on POA use during pregnancy and neurodevelopmental disorders to consider “as needed” prescriptions. Notably, we found comparable results when excluding “as needed” prescriptions and when using other indexes of POA exposure that examined different assumptions about the use of filled POAs during pregnancy. Taken together, these results help reduce the possibility that bias due to exposure misclassification accounts for the findings. We also triangulated findings from multiple approaches to target confounding that vary in their strengths and limitations. Our study also provides more information regarding the timing of POA exposure. Although some studies suggest exposure early in pregnancy is more highly associated with neuropsychiatric disorders [10], our findings are consistent with conclusions from previous studies that did not support greater risk for neurodevelopmental disorders during first-trimester exposure [9,12].

The results of our study have important translational implications. First, clinical decision-making for the treatment of pain during pregnancy requires birthing parents and physicians to weigh the risks and benefits of different analgesic medications [42], including dose or duration of medication exposure and stage of pregnancy [43]. Certainly, decisions to use POAs during pregnancy must be based on available data on the risks for multiple outcomes for the birthing parent and child(ren), including consideration of risks associated with poorly managed pain [43]. The present study suggests that a causal effect of moderate POA use during pregnancy on two common neurodevelopmental disorders in children, ASD and ADHD, may be minimal. Second, the findings of the current study suggest that research needs to explore the underlying mechanisms that are responsible for the increased risk for ASD and ADHD among those exposed to POAs during pregnancy, including the possible role of parental painful conditions and underlying pain pathophysiology [18,44,45].

There are several strengths of the current study. First, we utilized a national cohort of more than 1.2 million births. Second, we used recorded diagnoses of ASD after age two and of ADHD after age four to increase confidence in the validity of the diagnoses. Third, we used multiple quasi-experimental designs and found converging results across both models accounting for sources of measured and unmeasured confounding. Finally, we performed a variety of sensitivity analyses to evaluate the assumptions we made in our primary analysis and observed a similar pattern of results.

The results of this study must be interpreted considering several limitations. First, there are limitations to each of the designs. For example, our use of measured covariates is limited by our inability to account for many factors (e.g., illicit drug use by the birthing parent), measurement error in our covariates, and complex interactions between covariates [16]. Likewise, analyses restricting comparisons to those with painful conditions could increase bias due to unmeasured confounding [46], and sibling comparisons may be subject to carry-over effects (i.e., exposure in one pregnancy affecting a subsequent pregnancy) [47]. However, agreement across designs increases our confidence in the overall pattern of the findings. Second, there are limitations to relying on prescription information. For example, our indexes of POAs may not accurately capture true POA exposure, as we did not observe whether patients took their prescribed medications, potentially resulting in overly conservative estimates. Thus, the results are more analogous to intention to treat comparisons. Third, we were not able to capture over the counter use of paracetamol/acetaminophen or nonsteroidal anti-inflammatory drugs, though we were able to adjust for these and other prescribed pain medications. Fourth, given available statistical power, we cannot rule out small effects of POA exposure on these neurodevelopmental disorders.

Fifth, Swedish register data does not capture spontaneous abortions before 22 weeks gestation, and we do not have information on induced abortion. Thus, our analyses were restricted to live births. If POA exposure increases the likelihood of abortion and infants that would have later gone on to have ASD or ADHD are similarly less likely to survive pregnancy, the true association between POA exposure and risk for these neurodevelopmental disorders could be underestimated in this study [48–50]. However, the mechanism by which there would be additional spontaneous or induced abortion among fetuses that have a propensity for developing ASD or ADHD is unclear. Furthermore, previous research suggests that even when studying birth defects, an outcome where “livebirth bias” may be a significant concern, restricting to live births is unlikely to substantially alter results [51]. Sixth, while use of ICD-10 codes allows us to capture clinical diagnoses of ASD and ADHD, these codes may be subject to misclassification and may not capture all cases of ASD and ADHD in the population. Finally, future studies will need to explore the generalizability of the findings in samples with information on race/ethnicity, illicit substance use, and outside the medical and social context of Sweden.

We observed that children exposed to prenatal POAs have higher risk for ASD and ADHD, particularly with high dose and long duration of exposure, underscoring the need for caution when prescribing high doses. However, these associations were largely attenuated when accounting for factors associated with POA use in the year prior to conception and genetic and environmental factors shared by siblings. Thus, findings of the present study suggest that prenatal POA exposure, at the levels observed in our cohort, is not associated with a substantial increased risk for neurodevelopmental disorders. Rather, the findings indicate that the observed higher risks for ASD and ADHD were largely due to confounding, highlighting the importance of using multiple advanced designs to target unmeasured confounding when evaluating prenatal risk factors.

## Supporting information

S1 Fig Partial overlap with interval shift Δ. (DOCX)

S2 Fig Partial overlap without interval shift Δ. (DOCX)

S3 Fig Kaplan–Meier estimates of cumulative incidence for autism spectrum disorder by cumulative dose exposure level among children born to birthing parents with diagnosed painful conditions. (DOCX)

S4 Fig Kaplan–Meier estimates of cumulative incidence for autism spectrum disorder by cumulative dose exposure level among children born to birthing parents with POA use in the year before or during pregnancy. (DOCX)

S5 Fig Kaplan–Meier estimates of cumulative incidence for attention-deficit/hyperactivity disorder by cumulative dose exposure level among children born to birthing parents with diagnosed painful conditions. (DOCX)

S6 Fig Kaplan–Meier estimates of cumulative incidence for attention-deficit/hyperactivity disorder by cumulative dose exposure level among children born to birthing parents with POA use in the year before or during pregnancy. (DOCX)

S1 Table Sequential exclusions and information on follow-up time. (DOCX)

S2 Table Swedish registers. (DOCX)

S3 Table STROBE Statement—Checklist of items that should be included in reports of cohort studies. (DOCX)

S4 Table POA exposure based on N02A dispensations. (DOCX)

S5 Table Exposure to other psychoactive medications (birthing parent prescriptions) during pregnancy and in the year before conception. (DOCX)

S6 Table Psychiatric diagnoses. (DOCX)

S7 Table Pain indications. (DOCX)

S8 Table Distributions of number of prescriptions dispensed during pregnancy in analytic cohort. (DOCX)

S9 Table Distribution of duration of exposure in analytic cohort. (DOCX)

S10 Table Sample sizes and exposure distributions across models. (DOCX)

S11 Table Non-birthing parent characteristics. (DOCX)

S12 Table Background characteristics for dose and ASD sibling comparison. (DOCX)

S13 Table Sensitivity analyses (1 & 2) testing associations between duration of POA exposures and neurodevelopmental disorders. (DOCX)

S14 Table Sensitivity analysis 4 of dose and duration based on exposure in the first and second trimester of pregnancy only. (DOCX)

S15 Table Sensitivity analysis 5 of dose and duration based on maximum predicted use but no shifting of overlapping prescriptions. (DOCX)

S16 Table Sensitivity analysis 6 of continuous dose and duration of exposure. (DOCX)

S17 Table Sensitivity analysis 7 of sensitive periods of exposure and dose. (DOCX)

S18 Table Sensitivity analysis 7 of sensitive periods of exposure and duration. (DOCX)

S19 Table Sensitivity analysis 8 using covariates measured before conception only. (DOCX)

S20 Table Sensitivity analysis 9 using outcomes based on first ASD or ADHD diagnosis after birth. (DOCX)

S21 Table Parameters for Model 2 associations between dose and ASD and ADHD. (DOCX)

S1 Text Analysis plan and updates. (DOCX)

S2 Text Text-mining and exposure definitions. (DOCX)

S3 Text Trimester definitions. (DOCX)

S4 Text SAS 9.4 code for primary analyses and continuous sensitivity analyses. (DOCX)

## Abbreviations

ADHD: attention-deficit/hyperactivity disorder
ASD: autism spectrum disorder
ATC: Anatomical Therapeutic Chemical
CI: confidence interval
HRs: hazard ratios
ICD: International Classification of Diseases
IQR: interquartile range
KM: Kaplan–Meier
OME: oral morphine equivalent
POA: prescribed opioid analgesic
