# Supplementary figures and images for: Prescribed opioid analgesic use in pregnancy and risk of neurodevelopmental disorders in children: A retrospective study in Sweden
Source: PLoS Med. 2025 Sep 16;22(9):e1004721. doi: 10.1371/journal.pmed.1004721 (PMC12440195; doi:10.1371/journal.pmed.1004721)

**S1 Fig.** Partial overlap with interval shift Δ


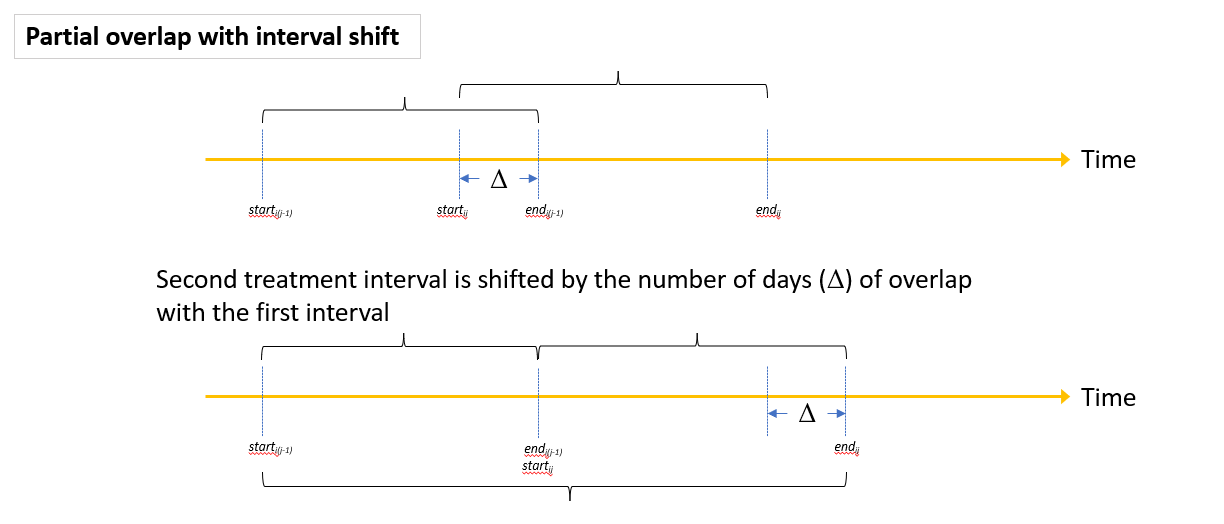

Supplement: S1 Fig — (DOCX) [file pmed.1004721.s001.docx]

**S2 Fig.** Partial overlap without interval shift Δ


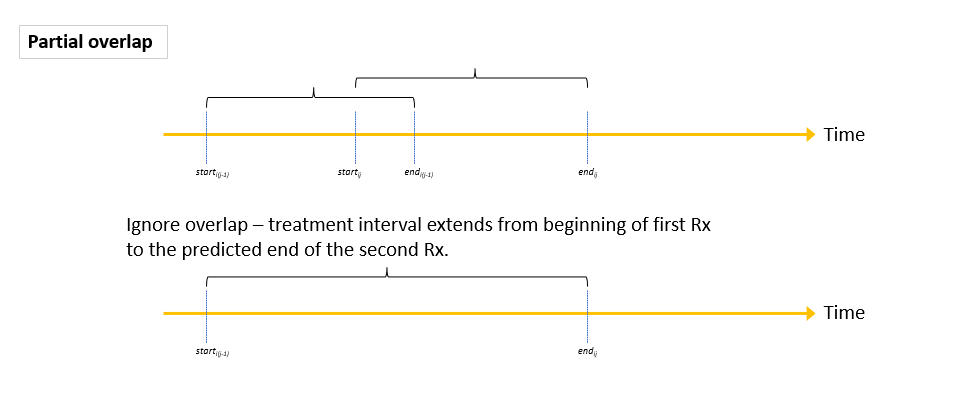

Supplement: S2 Fig — (DOCX) [file pmed.1004721.s002.docx]
