# Supplementary material for: Prescribed opioid analgesic use in pregnancy and risk of neurodevelopmental disorders in children: A retrospective study in Sweden
Source: PLoS Med. 2025 Sep 16;22(9):e1004721. doi: 10.1371/journal.pmed.1004721 (PMC12440195; doi:10.1371/journal.pmed.1004721)
Supplement: S3 Fig — (DOCX) [file pmed.1004721.s003.docx]

**S3 Fig.** Kaplan-Meier estimates of cumulative incidence for autism spectrum disorder by cumulative dose exposure level among children born to birthing parents with diagnosed painful conditions


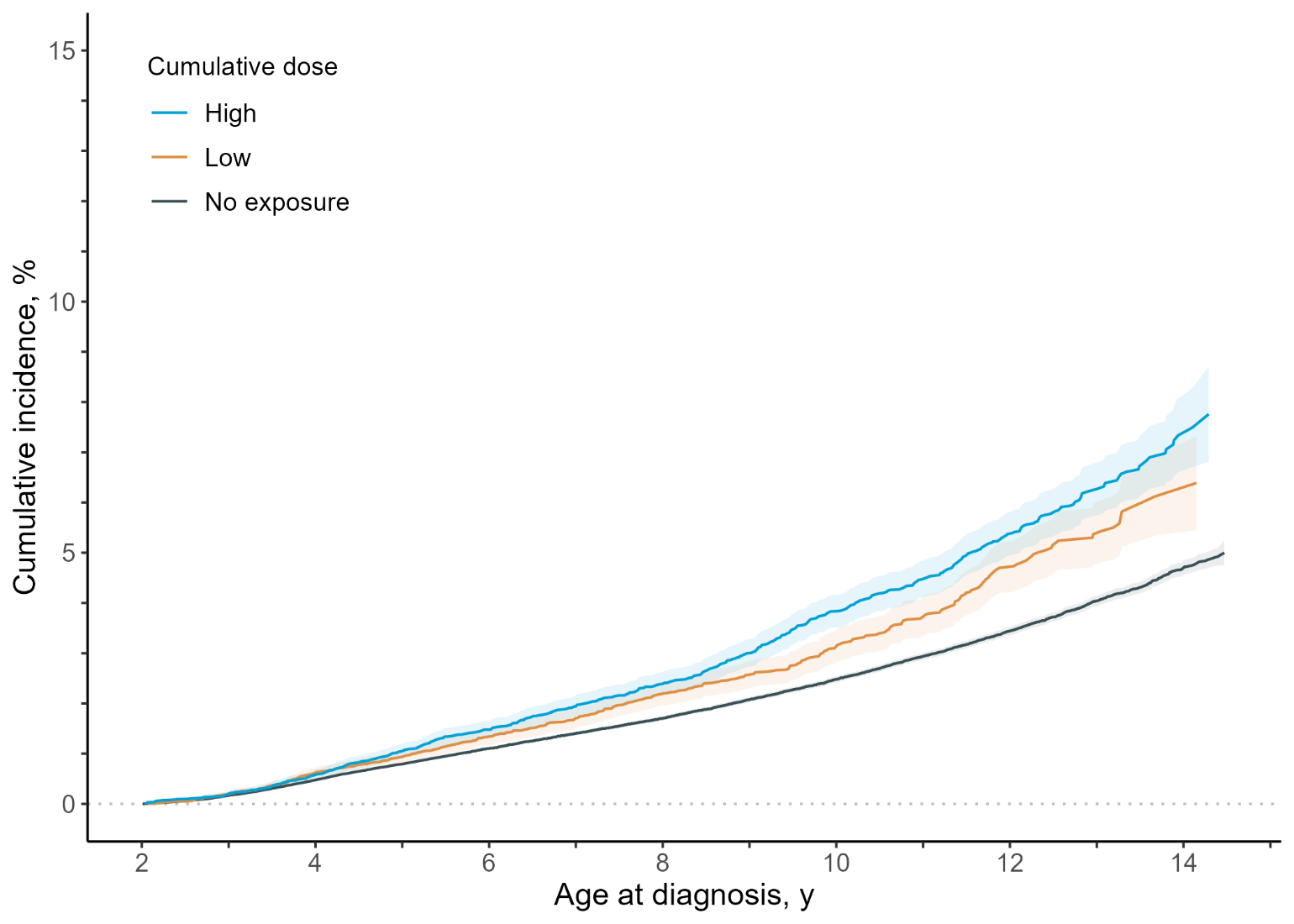


Note: Dose values are calculated based on daily maximum predicted use.
