# Supplementary material for: Prescribed opioid analgesic use in pregnancy and risk of neurodevelopmental disorders in children: A retrospective study in Sweden
Source: PLoS Med. 2025 Sep 16;22(9):e1004721. doi: 10.1371/journal.pmed.1004721 (PMC12440195; doi:10.1371/journal.pmed.1004721)
Supplement: S6 Fig — (DOCX) [file pmed.1004721.s006.docx]

**S6 Fig.** Kaplan-Meier estimates of cumulative incidence for attention-deficit/hyperactivity disorder by cumulative dose exposure level among children born to birthing parents with POA use in the year before or during pregnancy


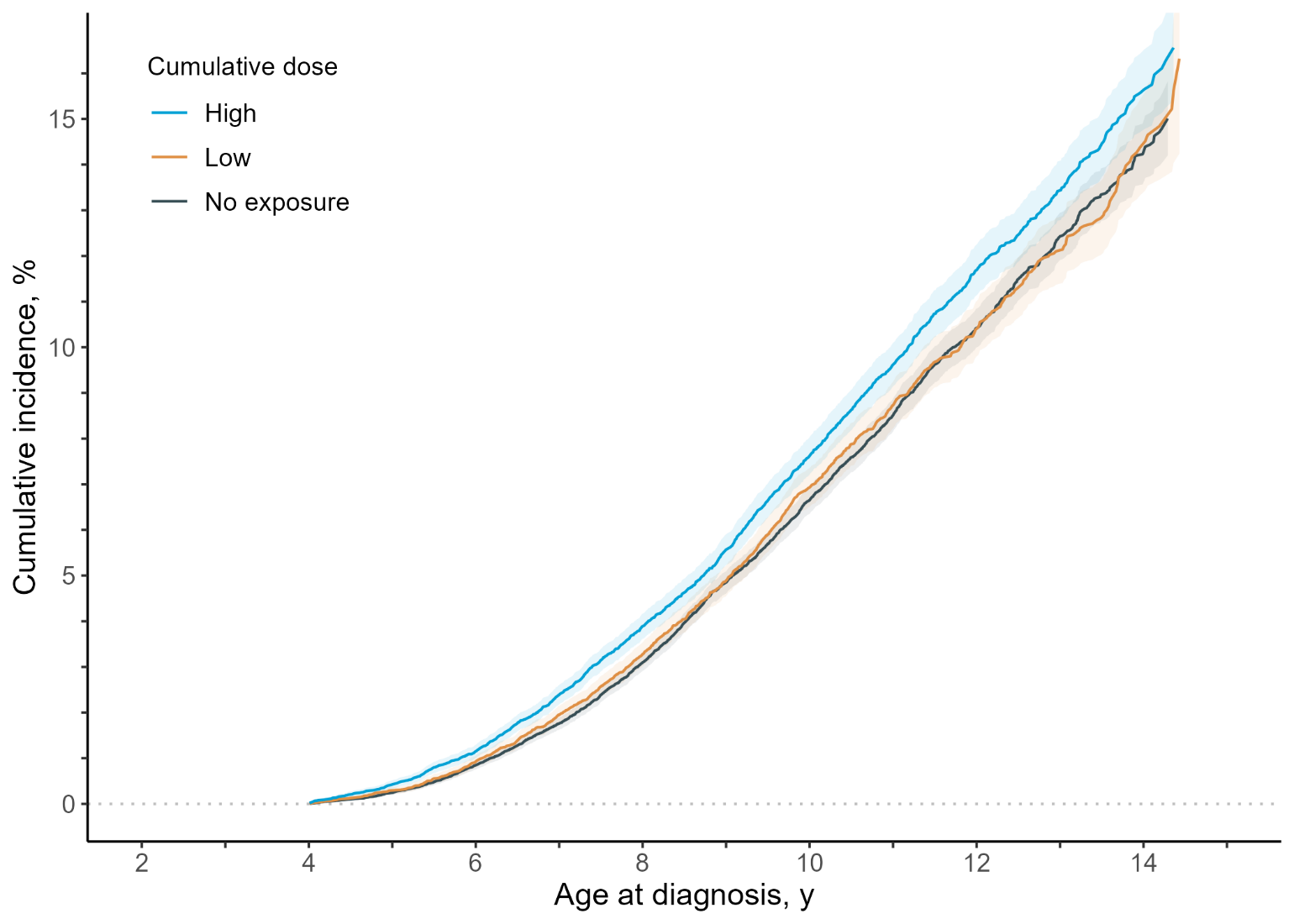


Note: Unexposed were children of individuals with any POA exposure during the year prior to conception but not during pregnancy, not dichotomized into high and low exposure. Dose values are calculated based on daily maximum predicted use.
