# Supplementary material for: Prescribed opioid analgesic use in pregnancy and risk of neurodevelopmental disorders in children: A retrospective study in Sweden
Source: PLoS Med. 2025 Sep 16;22(9):e1004721. doi: 10.1371/journal.pmed.1004721 (PMC12440195; doi:10.1371/journal.pmed.1004721)
Supplement: S1 Table — (DOCX) [file pmed.1004721.s007.docx]

**S1 Table.** Sequential exclusions and information on follow-up time

The end of study for all analyses was December 31st, 2021.To ensure that all included cases had at least 3 years of follow-up time and were at risk of being diagnosed with ASD, we only included births through December 31st, 2018. To allow for adequate follow-up time for diagnosis, all births in the ADHD model were required to have at least 6 years of follow-up time, thus models examining associations between exposure and ADHD were restricted to births from July 1st, 2007- December 31st, 2015. From the main cohort we excluded children who died or emigrated before age 2 and therefore would never be at risk of having either outcome by our definition. For ADHD models we further excluded cases who died or emigrated before age 4.

| Sequential exclusions |  |  |  |
| --- | --- | --- | --- |
| **ASD analysis** | **N dropped** | **N remaining** | **% included** |
| full cohort | 0 | 1287495 | 100 |
| stillbirths | 4780 | 1282715 | 99.6 |
| neonatal deaths | 1992 | 1280723 | 99.5 |
| deaths before age 2 | 1324 | 1279399 | 99.4 |
| emigration before age 2 | 8718 | 1270681 | 98.7 |
| invalid parity | 1 | 1270680 | 98.7 |
| invalid sex | 5 | 1270675 | 98.7 |
| Birthing parent opioid use disorder diagnosis before conception | 2275 | 1268400 | 98.5 |
| Birthing parent use of buprenorphine or methadone for opioid use disorder before or during pregnancy | 79 | 1268321 | 98.5 |
| Birthing parent use of naltrexone before or during pregnancy | 254 | 1268067 | 98.5 |
| only non-informative Rxs (Prescriptions lacking dosage information) | 43 | 1268024 | 98.5 |
| extreme OME values (top .1%) | 46 | 1267978 | 98.5 |
|  |  |  |  |
| **Additional exclusions for ADHD analysis** |  |  |  |
| only include births though December 31st, 2015 | 341899 | 926079 | 71.9 |
| deaths before age 4 | 183 | 925896 | 71.9 |
| emigration before age 4 | 7125 | 918771 | 71.4 |

Note: ASD analysis: median follow-up time 8.42 years, total cohort follow-up time: 10,792,794.21 years. ADHD analysis: median follow-up time 9.93 years, total cohort follow-up time 9,199,872.70 years. Abbreviation: oral morphine equivalent, OME.
