# Supplementary material for: Prescribed opioid analgesic use in pregnancy and risk of neurodevelopmental disorders in children: A retrospective study in Sweden
Source: PLoS Med. 2025 Sep 16;22(9):e1004721. doi: 10.1371/journal.pmed.1004721 (PMC12440195; doi:10.1371/journal.pmed.1004721)
Supplement: S2 Table — (DOCX) [file pmed.1004721.s008.docx]

| **S2 Table.** Swedish registers | |
| --- | --- |
| **Register** | **Information Contained** |
| The Multi-Generation Register | Biological relationships for all individuals born from 1932 and residing in Sweden since 1961. |
| The Prescribed Drug Register | Records of filled medication prescriptions since July 2005. |
| The Medical Birth Register | Information on 96-99% of births since 1973. |
| The National Patient Register | Diagnoses from all hospital admissions since 1987 and specialist outpatient care since 2001. |
| The National Crime Register | Criminal convictions since 1973. |
| The Education Register | Highest level of completed formal education since 1990. |
| The Integrated Database for Labor Market Research | Annual socio-economic data for all individuals since 1990. |
| The Cause of Death Register | Dates and causes of death since 1961 |
| Register of the Total Population | Dates of emigration out of Sweden since 1915 |
