# Supplementary material for: Prescribed opioid analgesic use in pregnancy and risk of neurodevelopmental disorders in children: A retrospective study in Sweden
Source: PLoS Med. 2025 Sep 16;22(9):e1004721. doi: 10.1371/journal.pmed.1004721 (PMC12440195; doi:10.1371/journal.pmed.1004721)
Supplement: S4 Table — (DOCX) [file pmed.1004721.s010.docx]

| **S4 Table.** POA exposure based on N02A dispensations | |
| --- | --- |
| **Anatomical therapeutic class codes** | **Medication** |
| N02AA01 | Morphine |
| N02AA03 | Hydromorphone |
| N02AA05 | Oxycodone |
| N02AA55 | Oxycodone/naloxone |
| N02AA59 | Codeine/paracetamol |
| N02AB01 | Ketobemidone |
| N02AB03 | Fentanyl |
| N02AC04 | Dextropropoxyphene |
| N02AE01 | Buprenorphine |
| N02AG01 | Morphine |
| N02AG02 | Ketobemidone/antispasmodics |
| N02AG04 | Hydromorphone/antispasmodics |
| N02AJ06 | Codeine/paracetamol |
| N02AJ08 | Codeine/ibuprofen |
| N02AJ09 | Codeine/other non-opioid analgesics |
| N02AX02 | Tramadol |
| N02AX06 | Tapentadol |
