# Supplementary material for: Prescribed opioid analgesic use in pregnancy and risk of neurodevelopmental disorders in children: A retrospective study in Sweden
Source: PLoS Med. 2025 Sep 16;22(9):e1004721. doi: 10.1371/journal.pmed.1004721 (PMC12440195; doi:10.1371/journal.pmed.1004721)
Supplement: S5 Table — (DOCX) [file pmed.1004721.s011.docx]

| **S5 Table.** Exposure to other psychoactive medications (birthing parent prescriptions) during pregnancy and in the year before conception | | |
| --- | --- | --- |
| ***Medication group*** | ***Anatomical therapeutic class codes*** | |
| Attention-deficit/hyperactivity disorder* | N06BA01, N06BA02, N06BA04, N06BA09, N06BA12 | |
| Benzodiazepine derivatives | N05BAxx, N05CDxx | |
| Benzodiazepine-related agents  (z- drugs) | N05CFxx | |
| Non-benzodiazepine anxiolytics | N05BBxx, N05BCxx, N05BDxx, N05BExx, N05BXxx | |
| Non-benzodiazepine hypnotics/sedatives | N05CAxx, N05CBxx, N05CCxx, N05CExx, N05CHxx, N05CMxx, N05CXxx | |
| Selective serotonin reuptake inhibitors & MAOIs, other antidepressants | N06ABxx, N06AFxx, N06AGxx, N06AXxx excluding N06AX21 | |
| Cyclic antidepressants | N06AAxx, N06ACxx, N06ADxx, N06AEx | |
| Antipsychotics | N05Axx excluding N05AN01 | |
| Anticonvulsants | N03Axx | |
| Mood stabilizer | N05AN01 | |
| Nicotine/alcohol use disorder | N07BAxx, N07BBxx (excluded naltrexone) | |
| Migraine | N02Cxx | |
| NSAIDs  Salicylic acid and derivatives | M01Axx, N02BA | |
| Paracetamol | N02BE01 | |
| Other pain meds | N06AX21, N02BF01, N02BF02 | |
| Exclusions:  Buprenorphine  Buprenorphine combinations  Methadone  Levomethadone  Naltrexone | | N07BC01  N07BC51  N07BC02  N07BC05  N07BB04 |

Note: *also used to identify child ADHD
