# Supplementary material for: Prescribed opioid analgesic use in pregnancy and risk of neurodevelopmental disorders in children: A retrospective study in Sweden
Source: PLoS Med. 2025 Sep 16;22(9):e1004721. doi: 10.1371/journal.pmed.1004721 (PMC12440195; doi:10.1371/journal.pmed.1004721)
Supplement: S6 Table — (DOCX) [file pmed.1004721.s012.docx]

| **S6 Table.** Psychiatric diagnoses | |
| --- | --- |
| **Diagnosis** | ***International Classification of Diseases* (ICD) Codes** |
| Autism spectrum disorder | Child outcome: F84 excluding F84.2 Rett’s syndrome  Parental covariate: ICD-10:F84 not including F84.2; ICD-9: 299 |
| Attention-deficit / hyperactivity disorder | Child outcome: F90  Parental covariate: ICD-10:F90, ICD-9: 314 |
| Opioid-related diagnoses | All F11 |
| Alcohol-related diagnoses | All F10 |
| Non-tobacco substance related diagnoses | All F12- F19 (except F17; tobacco) |
| Schizophrenia, schizotypal disorders, and delusional syndrome or bipolar disorder | All F20-F25, F28, F29, F30, F31 |
| Non-bipolar mood disorder | All F32-F34, F38, F39 |
| Anxiety disorders, OCD & trauma-related disorders | All F40-F43 |
| Definite or uncertain suicide attempt | All X60-X84, Y10-Y34  Specific codes: Y87.0, Y87.2 |
