# Supplementary material for: Prescribed opioid analgesic use in pregnancy and risk of neurodevelopmental disorders in children: A retrospective study in Sweden
Source: PLoS Med. 2025 Sep 16;22(9):e1004721. doi: 10.1371/journal.pmed.1004721 (PMC12440195; doi:10.1371/journal.pmed.1004721)
Supplement: S7 Table — (DOCX) [file pmed.1004721.s013.docx]

| **S7 Table.** Pain indications |  |
| --- | --- |
| **Category** | **ICD-10-SE codes** |
| **Musculoskeletal Disorders** |  |
| Arthritis/Joint disorders | M00-M25, R26 |
| Other musculoskeletal/ Connective tissue | M30-M36. M60-M94 |
| Back/neck disorders | M40-M54, Q76 |
| **Orofacial and headache disorders** |  |
| Migraine | G43 |
| Other headache syndromes | G44 |
| Headache | R51 |
| Temporomandibular joint disorders | K07.6 |
| Conditions of the teeth, mouth or jaw | K00-K14 |
| Eye pain | H57.1 |
| Ear pain | H92.0 |
| **Diseases of the urinary and genital organs** |  |
| Pain and other conditions associated with female genital organs and menstrual cycle | N71, N72, N73,N92 ,N94 |
| Endometriosis | N80 |
| Pain during intercourse | F52.5, F52.6 |
| Stones in urinary tract | N20-N23 |
| Interstitial cystitis (chronic) | N30.1 |
| Pain associated with micturition | R30 |
| **Pregnancy related** |  |
| Extra-uterine pregnancy | O00 |
| Complications from aborted pregnancy | O080, O081, O082, O083, O086, O087, O088, O089 |
| Symphyseolysis | O26.7 |
| Other pregnancy-related conditions | O268B, O268C, O268D |
| Anatomical challenges to labour/deliver | O33-O34 |
| **Functional disorders of the intestine** |  |
| Inflammatory bowel disease | K50, K51 |
| Irritable bowel syndrome | K58 |
| Other functional disorders of the intestine | K59 |
| Functional dyspepsia | K30 |
| Visceral pain | K40-46, K56, K57, K63, K65 |
| **Other pain-inducing conditions (excl. cancer)** |  |
| Neuropathy | B00-B09, G50, G54, G56, G57 |
| Polyneuropathies and other disorders of the peripheral nervous system | G60-64, G90, G99, B20-24, B25-B34 |
| Sickle-cell disorders | D57 |
| Diseases of the gallbladder, bile ducts and pancreas | K80, K81, K85, K86 |
| **Cancer and tumors** |  |
| Cancer | C00-C58, D00-D09 |
| Tumors | D10-D36, D37-D48 |
| **Not otherwise classified pain-defined diagnoses** |  |
| Pain in throat and chest | R07 |
| Pain from abdomen and pelvis | R10 |
| Other pain related codes | R52 |
| **Acute/injuries** |  |
| Injuries involving multiple body regions | T00-T07 |
| Injury to an unspecified part of the trunk, limb or other body region | T08-T14 |
| Effects of foreign body entering through natural orifice | T15-T19 |
| Burns and corrosions | T20-T32 |
| Frostbite | T33-T35 |
| Other and unspecified effects of external causes (e.g., radiation sickness unspecified, effects of heat and light, hypothermia) | T66-T78 |
| Early complications of injury by external violence (trauma) | T79 |
| Complications of surgical procedures and medical care not elsewhere classified | T80-T88 |
| Late complications of injuries, poisonings and other consequences of external causes | T90-T98 (excluding T96X) |
| **Injuries to a specified part of the body** | S00-S09, S10-S19, S20-S29, S30-S39, S40-S49, S50-S59  S60-S69, S70-S79, S80-S89, S90-S99 |
