# Supplementary material for: Prescribed opioid analgesic use in pregnancy and risk of neurodevelopmental disorders in children: A retrospective study in Sweden
Source: PLoS Med. 2025 Sep 16;22(9):e1004721. doi: 10.1371/journal.pmed.1004721 (PMC12440195; doi:10.1371/journal.pmed.1004721)
Supplement: S8 Table — (DOCX) [file pmed.1004721.s014.docx]

**S8 Table.** Distributions of number of prescriptions dispensed during pregnancy analytic cohort

| Number of Prescriptions dispensed during pregnancy | Frequency | % of total cohort |
| --- | --- | --- |
| 0 | 1,213,712 | 95.72% |
| 1 | 39,241 | 3.09% |
| 2 or more | 15,025 | 1.18% |

Note: This does not include prescriptions dispensed before conception that may overlap with the pregnancy interval
