# Supplementary material for: Prescribed opioid analgesic use in pregnancy and risk of neurodevelopmental disorders in children: A retrospective study in Sweden
Source: PLoS Med. 2025 Sep 16;22(9):e1004721. doi: 10.1371/journal.pmed.1004721 (PMC12440195; doi:10.1371/journal.pmed.1004721)
Supplement: S9 Table — (DOCX) [file pmed.1004721.s015.docx]

**S9 Table.** Distribution of duration of exposure in analytic cohort

| # of exposed days based on predicted daily maximum use | Frequency | % of total cohort |
| --- | --- | --- |
| 0 days | 1,212,551 | 95.63% |
| 1-7 days | 21,357 | 1.68% |
| 8-14 days | 13,206 | 1.04% |
| 15 + days | 20,864 | 1.65% |
