# Supplementary material for: Prescribed opioid analgesic use in pregnancy and risk of neurodevelopmental disorders in children: A retrospective study in Sweden
Source: PLoS Med. 2025 Sep 16;22(9):e1004721. doi: 10.1371/journal.pmed.1004721 (PMC12440195; doi:10.1371/journal.pmed.1004721)
Supplement: S10 Table — (DOCX) [file pmed.1004721.s016.docx]

**S10 Table.** Sample sizes and exposure distributions across models

|  | | | | | |
| --- | --- | --- | --- | --- | --- |
|  | **N (%)** | | | | |
|  | **1.Unadjusted** | **2.Covariate adjusted** | **3.Painful conditions** | **4.Before pregnancy** | **5.Sibling comparison*** |
| **Autism spectrum disorder (ASD)** | | | | | |
| Dose |  |  |  |  |  |
| Unexposed | 1,212,551 (95.6) | 1,212,551 (95.6) | 437,032 (91.4) | 52,031 (48.42) | 1414 (56.1) |
| Low | 29,185 (2.3) | 29,185 (2.3) | 20,756 (4.3) | 29,185 (27.2) | 571 (22.6) |
| High | 26,242 (2.1) | 26,242 (2.1) | 20,216 (4.2) | 26,242 (24.4) | 537 (21.3) |
|  |  |  |  |  |  |
| Duration |  |  |  |  |  |
| Unexposed | 1,212,551 (95.6) | 1,212,551 (95.6) | 437,032 (91.4) | 52,031 (48.4) | 1414 (55.2) |
| 1-7 days | 21,357 (1.7) | 21,357 (1.7) | 15,719 (3.3) | 21,357 (19.9) | 416 (16.2) |
| 8-14 days | 13,206 (1.0) | 13,206 (1.0) | 9,652 (2.0) | 13,206 (12.3) | 327 (12.8) |
| 15+ days | 20,864 (1.7) | 20,864 (1.7) | 15,601 (3.3) | 20,864 (19.4) | 405 (15.8) |
|  |  |  |  |  |  |
| **Attention-deficit/hyperactivity disorder (ADHD)** | | | | | |
| Dose |  |  |  |  |  |
| Unexposed | 877,510 (95.5) | 877,510 (95.5) | 308,203 (91.2) | 38,195 (48.1) | 2047 (52.4) |
| Low | 20,570 (2.2) | 20,570 (2.2) | 14,252 (4.2) | 20,570 (25.9) | 944 (24.2) |
| High | 20,691 (2.3) | 20,691 (2.3) | 15,679 (4.6) | 20,691 (26.0) | 915 (23.4) |
|  |  |  |  |  |  |
| Duration |  |  |  |  |  |
| Unexposed | 877,510 (95.5) | 877,510 (95.5) | 308,203 (91.2) | 38,195 (48.1) | 2047 (51.7) |
| 1-7 days | 15,128 (1.7) | 15,128 (1.7) | 10,840 (3.2) | 15,128 (19.0) | 670 (16.9) |
| 8-14 days | 10,130 (1.1) | 10,130 (1.1) | 7,306 (2.2) | 10,130 (12.8) | 507 (12.8) |
| 15+ days | 16,003 (1.7) | 16,003 (1.7) | 11,785 (3.5) | 16,003 (20.1) | 736 (18.6) |

Note: * Sample size presented here are for siblings that are discordant on both exposure and outcome status, thus samples differ for each model. ASD& OME: 2,522 siblings from 1072 families; ASD & duration: 2,562 siblings from 1,091 families; ADHD & OME: 3906 siblings from 1731 families; ADHD & duration: 3,960 siblings from 1759 families
