# Supplementary material for: Prescribed opioid analgesic use in pregnancy and risk of neurodevelopmental disorders in children: A retrospective study in Sweden
Source: PLoS Med. 2025 Sep 16;22(9):e1004721. doi: 10.1371/journal.pmed.1004721 (PMC12440195; doi:10.1371/journal.pmed.1004721)
Supplement: S11 Table — (DOCX) [file pmed.1004721.s017.docx]

| **S11 Table.** Non-birthing parent characteristics | | |  | |
| --- | --- | --- | --- | --- |
|  | **Unexposed** | | **Exposed** | |
|  | **N** | **%** | **N** | **%** |
|  | **1,212,551** | 95.6 | **55,427** | 4.4 |
| **Non-birthing parent characteristics** |  |  |  |  |
| Identity of non-birthing parent is known | 1,194,600 | 98.5 | 54,668 | 98.6 |
| Non-birthing parent information is missing | 17,951 | 1.5 | 759 | 1.4 |
| **Non-birthing parent age** |  |  |  |  |
| 19 or younger | 4,583 | 0.4 | 170 | 0.3 |
| 20-29 | 326,520 | 26.9 | 15,589 | 28.1 |
| 30-39 | 680,595 | 56.1 | 29,933 | 54.0 |
| 40-45 | 135,379 | 11.2 | 6,697 | 12.1 |
| 46 and older | 47,523 | 3.9 | 2,279 | 4.1 |
| **Non-birthing parent diagnoses before conception** | |  |  |  |
| Attention-deficit/hyperactivity disorder | 12,702 | 1.1 | 1,021 | 1.8 |
| Autism Spectrum disorder | 1,962 | 0.2 | 157 | 0.3 |
| Definite or uncertain suicide attempt | 24,652 | 2.0 | 1,593 | 2.9 |
| Opioid use disorder | 3,060 | 0.3 | 318 | 0.6 |
| Alcohol use disorder | 29,032 | 2.4 | 2,048 | 3.7 |
| Other non-tobacco substance use disorder | 15,301 | 1.3 | 1,362 | 2.5 |
| Serious mental illness | 6,828 | 0.6 | 483 | 0.9 |
| Non-bipolar mood disorder | 29,111 | 2.4 | 2,167 | 3.9 |
| Anxiety disorder | 45,740 | 3.8 | 3,115 | 5.6 |
| **Highest level of education** |  |  |  |  |
| Less than 9 years | 33,220 | 2.7 | 1,380 | 2.5 |
| 9 years | 110,358 | 9.1 | 6,654 | 12.0 |
| 1 to 3 years of upper secondary | 529,583 | 43.7 | 27,462 | 49.6 |
| Any post-secondary or postgraduate | 459,545 | 37.9 | 17,038 | 30.7 |
| Missing | 79,845 | 6.6 | 2,893 | 5.2 |
