# Supplementary material for: Prescribed opioid analgesic use in pregnancy and risk of neurodevelopmental disorders in children: A retrospective study in Sweden
Source: PLoS Med. 2025 Sep 16;22(9):e1004721. doi: 10.1371/journal.pmed.1004721 (PMC12440195; doi:10.1371/journal.pmed.1004721)
Supplement: S12 Table — (DOCX) [file pmed.1004721.s018.docx]

**S12 Table.** Background characteristics for dose and ASD sibling comparison

|  | **Total N= 2,522** | |
| --- | --- | --- |
|  | **N** | **%** |
| **Pregnancy-related characteristics** |  |  |
| **Birth order** |  |  |
| 1st | 784 | 31.1 |
| 2nd | 951 | 37.7 |
| 3rd | 434 | 17.2 |
| 4th or higher | 353 | 14.0 |
| **Year of birth** |  |  |
| 2007-2010 | 1080 | 42.8 |
| 2011-2014 | 1060 | 42.0 |
| 2015-2018 | 382 | 15.2 |
| **Female** | 940 | 37.3 |
| **Birthing parent smoking-3 months before pregnancy** | |  |
| None | 1744 | 69.2 |
| 1 to 9 cigarettes per day | 286 | 11.3 |
| 10 or more cigarettes per day | 388 | 15.4 |
| Missing | 104 | 4.1 |
| **Birthing parent smoking 1st trimester** |  |  |
| None | 2043 | 81.0 |
| 1 to 9 cigarettes per day | 287 | 11.4 |
| 10 or more cigarettes per day | 90 | 3.6 |
| Missing | 102 | 4.04 |
| **Exposure to other psychoactive medications** | |  |
| ADHD medication before | 41 | 1.6 |
| ADHD medication during | 18 | 0.7 |
| Anticonvulsants before | 93 | 3.7 |
| Anticonvulsants during | 43 | 1.7 |
| Lithium before | 15 | 0.6 |
| Lithium during | 6 | 0.2 |
| Antipsychotics excluding lithium before | 46 | 1.8 |
| Antipsychotics excluding lithium during | 25 | 1.0 |
| Non-benzodiazepine anxiolytics before | 147 | 5.8 |
| Non-benzodiazepine anxiolytics during | 31 | 1.2 |
| Benzodiazepine derivatives before | 144 | 5.7 |
| Benzodiazepine derivatives during | 50 | 2.0 |
| Benzodiazepine-related agents (z-drugs) before | 178 | 7.1 |
| Benzodiazepine-related agents (z-drugs) during | 64 | 2.5 |
| Cyclic antidepressants before | 44 | 1.7 |
| Cyclic antidepressants during | 13 | 0.5 |
| Non-benzodiazepine hypnotics/sedatives before | 109 | 4.3 |
| Non-benzodiazepine hypnotics/sedatives during | 88 | 3.5 |
| Migraine medications before | 125 | 5.0 |
| Migraine medications during | 69 | 2.7 |
| Medications for nicotine/alcohol use disorder before | 125 | 5.0 |
| Medications for nicotine/alcohol use disorder during | 1 | 0.0 |
| Other pain medications before | 23 | 0.9 |
| Other pain medications during | 10 | 0.4 |
| SSRIs before | 368 | 14.6 |
| SSRIs during | 210 | 8.3 |
| Paracetamol before | 395 | 15.7 |
| Paracetamol during | 357 | 14.2 |
| NSAIDs before | 559 | 22.2 |
| NSAIDs during | 104 | 4.1 |
| **Birthing parent characteristics** |  |  |
| **Birthing parent age** |  |  |
| 19 or younger | 60 | 2.4 |
| 20-29 | 1361 | 54.0 |
| 30-39 | 1048 | 41.6 |
| 40-45 | 48 | 1.9 |
| 46 and older | 5 | 0.2 |
| **Birthing parent diagnoses before conception** | |  |
| Attention-deficit/hyperactivity disorder | 86 | 3.4 |
| Autism Spectrum disorder | 26 | 1.0 |
| Definite or uncertain suicide attempt | 218 | 8.6 |
| Alcohol use disorder | 197 | 7.8 |
| Other non-tobacco substance use disorder | 64 | 2.5 |
| Serious mental illness | 64 | 2.5 |
| Non-bipolar mood disorder | 433 | 17.2 |
| Anxiety disorder | 503 | 19.9 |
| **Highest level of education** |  |  |
| Less than 9 years | 106 | 4.2 |
| 9 years | 526 | 20.9 |
| 1 to 3 years of upper secondary | 1030 | 40.8 |
| Any post-secondary or postgraduate | 728 | 28.9 |
| Missing | 132 | 5.2 |
| **Any pain conditions**** not a covariate* | 1720 | 68.2 |
| **Country of origin is Sweden** | 1910 | 75.7 |
| **Other familial and socioeconomic characteristics** | |  |
| **Cohabitation at Childbirth** |  |  |
| Cohabitating | 2153 | 85.4 |
| Single | 92 | 3.7 |
| Other cohabitation situation | 174 | 6.9 |
| Missing | 103 | 4.08 |
| **Birthing parent income in year before conception** | |  |
| 1st quintile (lowest) | 654 | 25.9 |
| 2nd quintile | 956 | 37.9 |
| 3rd quintile | 495 | 19.6 |
| 4th quintile | 228 | 9.0 |
| 5th quintile (highest) | 132 | 5.2 |
| Missing | 57 | 2.26 |

Note: This table includes siblings that were discordant on exposure and outcome status.

Abbreviations: POA, prescribed opioid analgesic; ADHD, attention-deficit/hyperactivity disorder;

SSRIs, selective serotonin reuptake inhibitors; NSAIDs, nonsteroidal anti-inflammatory drugs
