# Supplementary material for: Prescribed opioid analgesic use in pregnancy and risk of neurodevelopmental disorders in children: A retrospective study in Sweden
Source: PLoS Med. 2025 Sep 16;22(9):e1004721. doi: 10.1371/journal.pmed.1004721 (PMC12440195; doi:10.1371/journal.pmed.1004721)
Supplement: S13 Table — (DOCX) [file pmed.1004721.s019.docx]

| **S13 Table.** Sensitivity analyses (1 &2) testing associations between duration of POA exposures and neurodevelopmental disorders | | | | | |
| --- | --- | --- | --- | --- | --- |
|  | **HR 95% CI** |  |  |  |  |
|  | **1.Unadjusted** | **2. Covariate adjusted** | **3.Pain comparison** | **4.Before pregnancy** | **5.Sibling comparison** |
| **Autism spectrum disorder (ASD)** | | | | | |
| **Primary analysis results**: Duration | | | | | |
| Unexposed | Reference | Reference | Reference | Reference | Reference |
| 1-7 days | 1.42 (1.30, 1.55) | 1.22 (1.11, 1.33) | 1.12 (1.01, 1.24) | 0.99 (0.89, 1.10) | 1.06 (0.85, 1.33) |
| 8-14 days | 1.56 (1.41, 1.73) | 1.33 (1.20, 1.48) | 1.29 (1.15, 1.46) | 1.07 (0.95, 1.21) | 0.98 (0.76, 1.25) |
| 15+ days | 1.76 (1.63, 1.90) | 1.29 (1.19, 1.41) | 1.22 (1.10, 1.34) | 1.08 (0.97, 1.20) | 1.01 (0.81, 1.27) |
|  |  |  |  |  |  |
| **1**: Duration based on maximum use without the influence of as needed prescriptions in calculations | | | | | |
| Unexposed | Reference | Reference | Reference | Reference | Reference |
| 1-7 days | 1.46 (1.27, 1.69) | 1.23 (1.06, 1.42) | 1.11 (0.94, 1.32) | 0.99 (0.85, 1.15) | 1.24 (0.84, 1.83) |
| 8-14 days | 1.62 (1.39, 1.88) | 1.33 (1.14, 1.55) | 1.22 (1.02, 1.45) | 1.07 (0.91, 1.25) | 0.85 (0.59, 1.22) |
| 15+ days | 1.84 (1.64, 2.05) | 1.26 (1.12, 1.42) | 1.19 (1.04, 1.37) | 1.08 (0.96, 1.23) | 1.11 (0.81, 1.52) |
|  |  |  |  |  |  |
| **2:** Duration based on minimum predicted use | | | | | |
| Unexposed | Reference | Reference | Reference | Reference | Reference |
| 1-7 days | 1.94 (1.60, 2.34) | 1.59 (1.31, 1.92) | 1.41 (1.13, 1.76) | 1.30 (1.07, 1.58) | 1.35 (0.82, 2.24) |
| 8-14 days | 1.55 (1.28, 1.87) | 1.26 (1.04, 1.52) | 1.17 (0.95, 1.45) | 1.03 (0.85, 1.25) | 0.96 (0.60, 1.51) |
| 15+ days | 1.64 (1.50, 1.79) | 1.21 (1.10, 1.34) | 1.14 (1.02, 1.27) | 1.01 (0.92, 1.12) | 1.04 (0.80, 1.33) |
|  |  |  |  |  |  |
| **Attention-deficit/hyperactivity disorder (ADHD)** | | | | | |
| **Primary analysis results: Duration** | | | | | |
| Unexposed | Reference | Reference | Reference | Reference | Reference |
| 1-7 days | 1.61 (1.51, 1.72) | 1.29 (1.21, 1.37) | 1.21 (1.12, 1.30) | 1.01 (0.94, 1.09) | 1.07 (0.91, 1.27) |
| 8-14 days | 1.76 (1.64, 1.90) | 1.38 (1.28, 1.49) | 1.25 (1.15, 1.37) | 1.10 (1.01, 1.20) | 1.12 (0.92, 1.35) |
| 15+ days | 2.00 (1.89, 2.11) | 1.27 (1.20, 1.35) | 1.23 (1.15, 1.32) | 1.09 (1.01, 1.17) | 0.84 (0.71, 0.99) |
|  |  |  |  |  |  |
| **1**: Duration based on maximum use without the influence of as needed prescriptions in calculations | | | | | |
| Unexposed | Reference | Reference | Reference | Reference | Reference |
| 1-7 days | 1.71 (1.55, 1.88) | 1.28 (1.16, 1.42) | 1.19 (1.06, 1.34) | 1.04 (0.94, 1.15) | 1.19 (0.91, 1.55) |
| 8-14 days | 1.79 (1.61, 1.99) | 1.33 (1.19, 1.49) | 1.22 (1.07, 1.38) | 1.09 (0.98, 1.22) | 0.96 (0.74, 1.26) |
| 15+ days | 2.01 (1.86, 2.18) | 1.18 (1.09, 1.29) | 1.18 (1.07, 1.30) | 1.05 (0.96, 1.15) | 0.90 (0.71, 1.14) |
|  |  |  |  |  |  |
| **2:** Duration based on minimum predicted use | | | | | |
| Unexposed | Reference | Reference | Reference | Reference | Reference |
| 1-7 days | 1.97 (1.72, 2.26) | 1.45 (1.26, 1.67) | 1.32 (1.13, 1.55) | 1.17 (1.01, 1.34) | 1.43 (0.97, 2.12) |
| 8-14 days | 1.83 (1.61, 2.08) | 1.38 (1.21, 1.57) | 1.29 (1.11, 1.49) | 1.12 (0.98, 1.27) | 1.18 (0.85, 1.64) |
| 15+ days | 1.84 (1.73, 1.96) | 1.18 (1.10, 1.26) | 1.14 (1.05, 1.23) | 1.01 (0.94, 1.09) | 0.91 (0.76, 1.10) |
|  | | | | | |

Models 2-5 control for all variables listed in Table 1 and non-birthing parent characteristics listed in S11 Table.
