# Supplementary material for: Prescribed opioid analgesic use in pregnancy and risk of neurodevelopmental disorders in children: A retrospective study in Sweden
Source: PLoS Med. 2025 Sep 16;22(9):e1004721. doi: 10.1371/journal.pmed.1004721 (PMC12440195; doi:10.1371/journal.pmed.1004721)
Supplement: S14 Table — (DOCX) [file pmed.1004721.s020.docx]

**S14 Table.** Sensitivity analysis 4 of dose and duration based on exposure in the first and second trimester of pregnancy only

|  | **HR (95% CI)** | | | | |
| --- | --- | --- | --- | --- | --- |
|  | **1.Unadjusted** | **2.Covariate adjusted** | **3.Painful conditions** | **4.Before pregnancy** | **5.Sibling comparison** |
| **Autism spectrum disorder (ASD)** | | | | | |
| Dose |  |  |  |  |  |
| Unexposed | Reference | Reference | Reference | Reference | Reference |
| Low | 1.38 (1.25, 1.53) | 1.15 (1.04, 1.27) | 1.06 (0.94, 1.20) | 0.94 (0.84, 1.05) | 1.15 (0.90, 1.47) |
| High | 1.80 (1.65, 1.96) | 1.28 (1.17, 1.41) | 1.24 (1.12, 1.38) | 1.07 (0.97, 1.19) | 1.10 (0.86, 1.42) |
|  |  |  |  |  |  |
| Duration |  |  |  |  |  |
| Unexposed | Reference | Reference | Reference | Reference | Reference |
| 1-7 days | 1.36 (1.21, 1.53) | 1.14 (1.01, 1.28) | 1.05 (0.91, 1.20) | 0.92 (0.81, 1.04) | 1.39 (1.04, 1.87) |
| 8-14 days | 1.48 (1.29, 1.70) | 1.22 (1.06, 1.40) | 1.15 (0.98, 1.35) | 0.98 (0.85, 1.14) | 1.01 (0.74, 1.39) |
| 15+ days | 1.89 (1.72, 2.08) | 1.29 (1.16, 1.43) | 1.26 (1.12, 1.42) | 1.10 (0.98, 1.23) | 0.94 (0.74, 1.33) |
|  |  |  |  |  |  |
| **Attention-deficit/hyperactivity disorder (ADHD)** | | | | | |
| Dose |  |  |  |  |  |
| Unexposed | Reference | Reference | Reference | Reference | Reference |
| Low | 1.75 (1.63, 1.87) | 1.34 (1.25, 1.44) | 1.24 (1.14, 1.35) | 1.07 (0.99, 1.15) | 1.20 (1.00, 1.44) |
| High | 1.95 (1.83, 2.07) | 1.20 (1.12, 1.28) | 1.19 (1.10, 1.28) | 1.04 (0.96, 1.12) | 0.90 (0.75, 1.09) |
|  |  |  |  |  |  |
| Duration |  |  |  |  |  |
| Unexposed | Reference | Reference | Reference | Reference | Reference |
| 1-7 days | 1.62 (1.49, 1.75) | 1.24 (1.14, 1.34) | 1.17 (1.07, 1.29) | 0.98 (0.90, 1.07) | 1.33 (1.07, 1.65) |
| 8-14 days | 1.88 (1.72, 2.06) | 1.42 (1.29, 1.56) | 1.34 (1.20, 1.49) | 1.14 (1.04, 1.26) | 1.03 (0.81, 1.31) |
| 15+ days | 2.04 (1.91, 2.19) | 1.19 (1.11, 1.29) | 1.18 (1.08, 1.28) | 1.06 (0.97, 1.15) | 0.83 (0.67, 1.02) |

Models 2-5 control for all variables listed in Table 1 and non-birthing parent characteristics listed in S11 Table.
