# Supplementary material for: Prescribed opioid analgesic use in pregnancy and risk of neurodevelopmental disorders in children: A retrospective study in Sweden
Source: PLoS Med. 2025 Sep 16;22(9):e1004721. doi: 10.1371/journal.pmed.1004721 (PMC12440195; doi:10.1371/journal.pmed.1004721)
Supplement: S15 Table — (DOCX) [file pmed.1004721.s021.docx]

**S15 Table.** Sensitivity analysis 5 of dose and duration based on maximum predicted use but no shifting of overlapping prescriptions

|  | **HR 95% CI** | |  | |  | |  | |  | |
| --- | --- | --- | --- | --- | --- | --- | --- | --- | --- | --- |
|  | **1.Unadjusted** | | **2. Covariate adjusted** | | **3.Pain comparison** | | **4.Before pregnancy** | | **5.Sibling comparison** | |
| **Autism spectrum disorder (ASD)** | | | | | | | | | | |
| Dose | | | | | | | | | | |
| Unexposed | | Reference | | Reference | | Reference | | Reference | | Reference |
| Low | | 1.42 (1.31, 1.54) | | 1.21 (1.12, 1.31) | | 1.14 (1.04, 1.25) | | 0.99 (0.90, 1.09) | | 1.05 (0.87, 1.27) |
| High | | 1.74 (1.63, 1.87) | | 1.34 (1.24, 1.44) | | 1.25 (1.15, 1.36) | | 1.10 (1.00, 1.21) | | 0.99 (0.81, 1.21) |
|  |  | |  | |  | |  | |  | |
| Duration | | | | | | | | | | |
| Unexposed | Reference | | Reference | | Reference | | Reference | | Reference | |
| 1-7 days | 1.43 (1.31, 1.56) | | 1.23 (1.12, 1.34) | | 1.13 (1.02, 1.25) | | 1.00 (0.90, 1.11) | | 1.06 (0.85, 1.32) | |
| 8-14 days | 1.56 (1.41, 1.73) | | 1.32 (1.19, 1.47) | | 1.30 (1.15, 1.46) | | 1.06 (0.94, 1.20) | | 0.98 (0.77, 1.26) | |
| 15+ days | 1.73 (1.61, 1.87) | | 1.29 (1.19, 1.40) | | 1.21 (1.10, 1.33) | | 1.06 (0.96, 1.17) | | 0.98 (0.79, 1.22) | |
|  |  | |  | |  | |  | |  | |
| **Attention-deficit/hyperactivity disorder (ADHD)** | | | | | | | | | | |
| Dose | | | | | | | | | | |
| Unexposed | | Reference | | Reference | | Reference | | Reference | | Reference |
| Low | | 1.71 (1.62, 1.81) | | 1.35 (1.28, 1.43) | | 1.25 (1.17, 1.34) | | 1.07 (1.00, 1.14) | | 1.04 (0.90, 1.19) |
| High | | 1.89 (1.80, 1.98) | | 1.26 (1.19, 1.33) | | 1.21 (1.14, 1.29) | | 1.06 (0.99, 1.14) | | 0.94 (0.81, 1.09) |
|  | | | | | | | | | | |
| Duration | | | | | | | | | | |
| Unexposed | Reference | | Reference | | Reference | | Reference | | Reference | |
| 1-7 days | 1.61 (1.51, 1.72) | | 1.29 (1.21, 1.38) | | 1.21 (1.12, 1.31) | | 1.01 (0.94, 1.09) | | 1.06 (0.90, 1.25) | |
| 8-14 days | 1.75 (1.63, 1.88) | | 1.36 (1.26, 1.47) | | 1.24 (1.14, 1.35) | | 1.08 (1.00, 1.18) | | 1.14 (0.94, 1.39) | |
| 15+ days | 1.96 (1.86, 2.06) | | 1.27 (1.19, 1.34) | | 1.22 (1.14, 1.31) | | 1.07 (1.00, 1.15) | | 0.88 (0.75, 1.04) | |

Models 2-5 control for all variables listed in Table 1 and non-birthing parent characteristics listed in S11 Table.
