# Supplementary material for: Prescribed opioid analgesic use in pregnancy and risk of neurodevelopmental disorders in children: A retrospective study in Sweden
Source: PLoS Med. 2025 Sep 16;22(9):e1004721. doi: 10.1371/journal.pmed.1004721 (PMC12440195; doi:10.1371/journal.pmed.1004721)
Supplement: S16 Table — (DOCX) [file pmed.1004721.s022.docx]

**S16 Table.** Sensitivity analysis 6 of continuous dose and duration of exposure

|  | **HR (95% CI)** | | | | |
| --- | --- | --- | --- | --- | --- |
|  | **1.Unadjusted** | **2.Covariate adjusted** | **3.Painful conditions** | **4.Before pregnancy** | **5.Sibling comparison** |
| **Autism spectrum disorder (ASD)** | | | | | |
| Dose (/100mg) |  |  |  |  |  |
|  | 1.009 (1.008, 1.011) | 1.002 (1.000, 1.005) | 1.002 (1.000, 1.005) | 1.002 (0.999, 1.004) | 1.002 (0.989, 1.014) |
|  |  |  |  |  |  |
| Duration (/7 days) |  |  |  |  |  |
|  | 1.036 (1.032, 1.041) | 1.012 (1.006, 1.018) | 1.010 (1.003, 1.016) | 1.005 (0.998, 1.012) | 0.987 (0.965, 1.010) |
|  |  |  |  |  |  |
| **Attention-deficit/hyperactivity disorder (ADHD)** | | | | | |
| Dose (/100mg) |  |  |  |  |  |
|  | 1.011 (1.010, 1.012) | 1.001 (0.999, 1.003) | 1.001 (0.999, 1.003) | 1.001 (0.999, 1.002) | 0.996 (0.988, 1.004) |
|  |  |  |  |  |  |
| Duration (/7 days) |  |  |  |  |  |
|  | 1.043 (1.039, 1.046) | 1.007 (1.003, 1.012) | 1.007 (1.002, 1.012) | 1.003 (0.998, 1.008) | 0.986 (0.969, 1.002) |

Models 2-5 control for all variables listed in Table 1 and non-birthing parent characteristics listed in S11 Table.
