# Supplementary material for: Prescribed opioid analgesic use in pregnancy and risk of neurodevelopmental disorders in children: A retrospective study in Sweden
Source: PLoS Med. 2025 Sep 16;22(9):e1004721. doi: 10.1371/journal.pmed.1004721 (PMC12440195; doi:10.1371/journal.pmed.1004721)
Supplement: S17 Table — (DOCX) [file pmed.1004721.s023.docx]

**S17 Table.** Sensitivity analysis 7 of sensitive periods of exposure and dose

|  | **3.Painful conditions** | **4.Before pregnancy** |
| --- | --- | --- |
| **Autism spectrum disorder (ASD)** | | |
| **Dose** |  |  |
| Unexposed | Reference | Reference |
| Low |  |  |
| Early | 1.11 (0.89, 1.37) | 0.99 (0.82, 1.19) |
| Middle/Late | 1.13 (1.02, 1.26) | 0.97 (0.87, 1.08) |
| High |  |  |
| Early | 1.20 (0.98, 1.47) | 1.04 (0.86, 1.26) |
| Middle/Late | 1.21 (1.09, 1.35) | 1.06 (0.95, 1.19) |
|  |  |  |
| **Attention-deficit/hyperactivity disorder (ADHD)** | | |
| **Dose** |  |  |
| Unexposed | Reference | Reference |
| Low |  |  |
| Early | 1.24 (1.06, 1.45) | 1.10 (0.96, 1.25) |
| Middle/Late | 1.25 (1.16, 1.34) | 1.05 (0.98, 1.14) |
| High |  |  |
| Early | 1.04 (0.89, 1.20)* | 0.92 (0.80, 1.05)* |
| Middle/Late | 1.23 (1.14, 1.32)* | 1.07 (0.99, 1.16)* |

Note: early exposure is exposure in the first trimester only. Middle/Late exposure is exposure in the second or third trimester only. *Indicates statistically significant differences (p<.05) between early and middle/late exposure. Models 3&4 control for all variables listed in Table 1 and non-birthing parent characteristics listed in S11 Table.
