# Supplementary material for: Prescribed opioid analgesic use in pregnancy and risk of neurodevelopmental disorders in children: A retrospective study in Sweden
Source: PLoS Med. 2025 Sep 16;22(9):e1004721. doi: 10.1371/journal.pmed.1004721 (PMC12440195; doi:10.1371/journal.pmed.1004721)
Supplement: S18 Table — (DOCX) [file pmed.1004721.s024.docx]

**S18 Table.** Sensitivity analysis 7 of sensitive periods of exposure and duration

|  | **3.Painful conditions** | **4.Before pregnancy** |
| --- | --- | --- |
| **Autism spectrum disorder (ASD)** | | |
| **Duration** |  |  |
| Unexposed | Reference | Reference |
| 1-7 days |  |  |
| Early | 0.99 (0.78, 1.26) | 0.94 (0.77, 1.16) |
| Middle/Late | 1.14 (1.02, 1.28) | 0.98 (0.87, 1.10) |
| 8-14 days |  |  |
| Early | 1.41 (1.08, 1.84) | 1.14 (0.89, 1.45) |
| Middle/Late | 1.29 (1.13, 1.48) | 1.07 (0.94, 1.22) |
| 15+ days |  |  |
| Early | 1.17 (0.91, 1.51) | 1.01 (0.80, 1.27) |
| Middle/Late | 1.11 (0.98, 1.27) | 1.01 (0.89, 1.14) |
|  | | |
| **Attention-deficit/hyperactivity disorder (ADHD)** | | |
| **Duration** |  |  |
| Unexposed | Reference | Reference |
| 1-7 days |  |  |
| Early | 1.14 (0.96, 1.35) | 1.00 (0.87, 1.16) |
| Middle/Late | 1.21 (1.11, 1.32) | 1.01 (0.93, 1.10) |
| 8-14 days |  |  |
| Early | 1.23 (1.00, 1.50) | 1.07 (0.90, 1.27) |
| Middle/Late | 1.24 (1.13, 1.37) | 1.10 (1.01, 1.21) |
| 15+ days |  |  |
| Early | 1.04 (0.86, 1.26) | 0.95 (0.81, 1.12) |
| Middle/Late | 1.26 (1.16, 1.37) | 1.09 (1.00, 1.19) |

Note: early exposure is exposure in the first trimester only. Middle/Late exposure is exposure in the second or third trimester only. *Indicates statistically significant differences (p<.05) between early and middle/late exposure. Models 3 & 4 control for all variables listed in Table 1 and non-birthing parent characteristics listed in S11 Table.
