# Supplementary material for: Prescribed opioid analgesic use in pregnancy and risk of neurodevelopmental disorders in children: A retrospective study in Sweden
Source: PLoS Med. 2025 Sep 16;22(9):e1004721. doi: 10.1371/journal.pmed.1004721 (PMC12440195; doi:10.1371/journal.pmed.1004721)
Supplement: S19 Table — (DOCX) [file pmed.1004721.s025.docx]

**S19 Table.** Sensitivity analysis 8 using covariates measured before conception only

|  | **HR (95% CI)** | | | | |
| --- | --- | --- | --- | --- | --- |
|  | **1.Unadjusted** | **2.Covariate adjusted** | **3.Painful conditions** | **4.Before pregnancy** | **5.Sibling comparison** |
| **Autism spectrum disorder (ASD)** | | | | | |
| Dose |  |  |  |  |  |
| Unexposed | Reference | Reference | Reference | Reference | Reference |
| Low | 1.42 (1.31, 1.54) | 1.22 (1.13, 1.32) | 1.15 (1.05, 1.26) | 1.00 (0.91, 1.10) | 1.04 (0.86, 1.26) |
| High | 1.74 (1.63, 1.87) | 1.37 (1.28, 1.47) | 1.27 (1.17, 1.38) | 1.13 (1.03, 1.23) | 0.96 (0.79, 1.17) |
|  |  |  |  |  |  |
| Duration |  |  |  |  |  |
| Unexposed | Reference | Reference | Reference | Reference | Reference |
| 1-7 days | 1.42 (1.30, 1.55) | 1.23 (1.12, 1.34) | 1.12 (1.01, 1.25) | 1.00 (0.90, 1.11) | 1.05 (0.84, 1.31) |
| 8-14 days | 1.56 (1.41, 1.73) | 1.35 (1.22, 1.50) | 1.31 (1.16, 1.47) | 1.09 (0.97, 1.23) | 0.96 (0.75, 1.23) |
| 15+ days | 1.76 (1.63, 1.90) | 1.33 (1.23, 1.44) | 1.24 (1.13, 1.36) | 1.11 (1.01, 1.22) | 0.99 (0.79, 1.24) |
|  |  |  |  |  |  |
| **Attention-deficit/hyperactivity disorder (ADHD)** | | | | | |
| Dose |  |  |  |  |  |
| Unexposed | Reference | Reference | Reference | Reference | Reference |
| Low | 1.71 (1.62, 1.81) | 1.38 (1.30, 1.45) | 1.27 (1.19, 1.35) | 1.08 (1.01, 1.15) | 1.04 (0.90, 1.19) |
| High | 1.89 (1.80, 1.98) | 1.32 (1.25, 1.39) | 1.26 (1.19, 1.33) | 1.09 (1.03, 1.16) | 0.94 (0.81, 1.008) |
|  |  |  |  |  |  |
| Duration |  |  |  |  |  |
| Unexposed | Reference | Reference | Reference | Reference | Reference |
| 1-7 days | 1.61 (1.51, 1.72) | 1.31 (1.23, 1.40) | 1.22 (1.13, 1.32) | 1.02 (0.95, 1.10) | 1.06 (0.90, 1.26) |
| 8-14 days | 1.76 (1.64, 1.90) | 1.42 (1.32, 1.53) | 1.28 (1.18, 1.40) | 1.12 (1.03, 1.22) | 1.11 (0.92, 1.35) |
| 15+ days | 2.00 (1.89, 2.11) | 1.33 (1.25, 1.41) | 1.28 (1.20, 1.37) | 1.12 (1.04, 1.20) | 0.84 (0.71, 0.99) |
| Models 2-5 control for all variables listed in Table 1 and non-birthing parent characteristics listed in S11 Table. | | | | | |
