# Supplementary material for: Prescribed opioid analgesic use in pregnancy and risk of neurodevelopmental disorders in children: A retrospective study in Sweden
Source: PLoS Med. 2025 Sep 16;22(9):e1004721. doi: 10.1371/journal.pmed.1004721 (PMC12440195; doi:10.1371/journal.pmed.1004721)
Supplement: S20 Table — (DOCX) [file pmed.1004721.s026.docx]

**S20 Table.** Sensitivity analysis 9 using outcomes based on first ASD or ADHD diagnosis after birth

In the primary analysis, we only considered the first ASD diagnosis made after age 2 (n=22,812 after sequential exclusions) and the first ADHD diagnosis made after age 4 (n=41,768) to improve confidence in diagnosis validity and stability.

To address potential bias resulting from exclusion of cases that were not eligible to have a diagnosis, we completed a sensitivity analysis in which we used the first ASD or ADHD diagnosis/medication dispensation after birth.

- The earliest ASD diagnosis was at just over 1 month old and the earliest ADHD diagnosis/medication dispensation was at 5.5 months old.
- There were 71 children who received an ASD diagnosis before age 2 who did not receive any subsequent diagnoses after age 2.
- There were 100 children who received an ADHD diagnosis before age 4 who did not receive any diagnoses after age 4 (births through December 31^st^, 2015 only).

|  | **HR (95% CI)** | | | | |
| --- | --- | --- | --- | --- | --- |
|  | **1.Unadjusted** | **2.Covariate adjusted** | **3.Painful conditions** | **4.Before pregnancy** | **5.Sibling comparison** |
| **Autism spectrum disorder (ASD)** | | | | | |
| Dose |  |  |  |  |  |
| Unexposed | Reference | Reference | Reference | Reference | Reference |
| Low | 1.42 (1.31, 1.53) | 1.21 (1.12, 1.31) | 1.14 (1.04, 1.25) | 0.99 (0.90, 1.09) | 1.05 (0.87, 1.27) |
| High | 1.73 (1.62, 1.86) | 1.33 (1.23, 1.43) | 1.25 (1.14, 1.36) | 1.10 (1.00, 1.21) | 0.99 (0.81, 1.21) |
|  |  |  |  |  |  |
| Duration |  |  |  |  |  |
| Unexposed | Reference | Reference | Reference | Reference | Reference |
| 1-7 days | 1.42 (1.29, 1.55) | 1.21 (1.11, 1.33) | 1.12 (1.01, 1.24) | 0.99 (0.89, 1.10) | 1.06 (0.85, 1.32) |
| 8-14 days | 1.56 (1.41, 1.73) | 1.33 (1.19, 1.47 | 1.29 (1.14, 1.45) | 1.07 (0.95, 1.21) | 0.98 (0.76, 1.25) |
| 15+ days | 1.75 (1.62, 1.89) | 1.29 (1.18, 1.40) | 1.21 (1.10, 1.33) | 1.08 (0.97, 1.19) | 1.01 (0.81, 1.27) |
|  |  |  |  |  |  |
| **Attention-deficit/hyperactivity disorder (ADHD)** | | | | | |
| Dose |  |  |  |  |  |
| Unexposed | Reference | Reference | Reference | Reference | Reference |
| Low | 1.71 (1.62, 1.80) | 1.35 (1.28, 1.43) | 1.25 (1.17, 1.33) | 1.06 (0.99, 1.14) | 1.04 (0.90, 1.19) |
| High | 1.88 (1.80, 1.98) | 1.26 (1.20, 1.33) | 1.21 (1.14, 1.29) | 1.06 (0.99, 1.14) | 0.94 (0.81, 1.09) |
|  |  |  |  |  |  |
| Duration |  |  |  |  |  |
| Unexposed | Reference | Reference | Reference | Reference | Reference |
| 1-7 days | 1.62 (1.52, 1.72) | 1.29 (1.21, 1.38) | 1.21 (1.12, 1.30) | 1.01 (0.94, 1.09) | 1.07 (0.91, 1.27) |
| 8-14 days | 1.76 (1.64, 1.89) | 1.38 (1.28, 1.49) | 1.25 (1.15, 1.36) | 1.10 (1.01, 1.20) | 1.11 (0.92, 1.35) |
| 15+ days | 1.99 (1.89, 2.11) | 1.27 (1.19, 1.35) | 1.23 (1.14, 1.31) | 1.08 (1.01, 1.16) | 0.84 (0.71, 0.99) |

Models 2-5 control for all variables listed in Table 1 and non-birthing parent characteristics listed in S11 Table.
