# Supplementary material for: Prescribed opioid analgesic use in pregnancy and risk of neurodevelopmental disorders in children: A retrospective study in Sweden
Source: PLoS Med. 2025 Sep 16;22(9):e1004721. doi: 10.1371/journal.pmed.1004721 (PMC12440195; doi:10.1371/journal.pmed.1004721)
Supplement: S21 Table — (DOCX) [file pmed.1004721.s027.docx]

**S21 Table.** Parameters for Model 2 associations between dose and ASD and ADHD

|  | **ASD** | **ADHD** | | |
| --- | --- | --- | --- | --- |
|  | **HR 95% CI** | **HR 95% CI** | | |
| **Pregnancy-related characteristics** |  |  | | |
| **Birth order** |  |  | | |
| 1st | *Reference* | *Reference* | | |
| 2nd | 0.69 (0.67, 0.71) | 0.97 (0.95, 0.99) | | |
| 3rd | 0.66 (0.63, 0.69) | 1.04 (1.01, 1.07) | | |
| 4th or higher | 0.74 (0.70, 0.79) | 1.14 (1.09, 1.19) | | |
| **Year of birth** |  |  | | |
| 2007-2010 | *Reference* | *Reference* | | |
| 2011-2014 | 1.15 (1.12, 1.19) | 1.11 (1.09, 1.14) | | |
| 2015-2018 | 1.44 (1.38, 1.51) | 1.07 (0.99, 1.15) | | |
| **Multiple births** | 1.35 (1.24, 1.47) | 1.08 (1.01, 1.15) | | |
| **Female** | 0.35 (0.34, 0.36) | 0.39 (0.38, 0.40) | | |
| **Birthing parent smoking-3 months before pregnancy** | | |  |  |
| None | *Reference* | *Reference* | | |
| 1 to 9 cigarettes per day | 1.05 (1.00, 1.11) | 1.28 (1.24, 1.33) | | |
| 10 or more cigarettes per day | 1.12 (1.05, 1.19) | 1.40 (1.35, 1.46) | | |
| **Birthing parent smoking 1st trimester** |  |  | | |
| None | *Reference* | *Reference* | | |
| 1 to 9 cigarettes per day | 1.12 (1.04, 1.20) | 1.17 (1.12, 1.22) | | |
| 10 or more cigarettes per day | 1.25 (1.13, 1.39) | 1.18 (1.10, 1.26) | | |
| **Exposure to other psychoactive medications** |  |  | | |
| ADHD medication before | 1.05 (0.86, 1.29) | 1.14 (0.98, 1.32) | | |
| ADHD medication during | 0.92 (0.71, 1.20) | 0.75 (0.61, 0.92) | | |
| Anticonvulsants before | 1.24 (1.10, 1.41) | 0.99 (0.89, 1.10) | | |
| Anticonvulsants during | 0.98 (0.83, 1.17) | 1.01 (0.88, 1.17) | | |
| Lithium before | 1.19 (0.81, 1.75) | 1.11 (0.78, 1.59) | | |
| Lithium during | 1.41 (0.87, 2.29) | 1.52 (0.95, 2.43) | | |
| Antipsychotics excluding lithium before | 0.90 (0.77, 1.06) | 0.93 (0.81, 1.06) | | |
| Antipsychotics excluding lithium during | 1.01 (0.82, 1.24) | 0.80 (0.66, 0.97) | | |
| Non-benzodiazepine anxiolytics before | 1.11 (1.03, 1.20) | 1.07 (1.01, 1.14) | | |
| Non-benzodiazepine anxiolytics during | 1.05 (0.89, 1.24) | 0.99 (0.87, 1.13) | | |
| Benzodiazepine derivatives before | 1.15 (1.05, 1.26) | 1.04 (0.97, 1.11) | | |
| Benzodiazepine derivatives during | 0.88 (0.75, 1.02) | 0.92 (0.82, 1.03) | | |
| Benzodiazepine-related agents (z-drugs) before | 1.07 (0.99, 1.17) | 1.10 (1.04, 1.17) | | |
| Benzodiazepine-related agents (z-drugs) during | 0.89 (0.77, 1.02) | 0.92 (0.83, 1.02) | | |
| Cyclic antidepressants before | 1.13 (0.97, 1.32) | 1.02 (0.90, 1.15) | | |
| Cyclic antidepressants during | 1.15 (0.87, 1.52) | 0.95 (0.75, 1.21) | | |
| Non-benzodiazepine hypnotics/sedatives before | 1.08 (0.99, 1.19) | 1.05 (0.97, 1.13) | | |
| Non-benzodiazepine hypnotics/sedatives during | 1.05 (0.94, 1.17) | 1.01 (0.92, 1.10) | | |
| Migraine medications before | 1.09 (0.99, 1.21) | 1.10 (1.02, 1.18) | | |
| Migraine medications during | 1.12 (0.97, 1.30) | 1.08 (0.97, 1.21) | | |
| Medications for nicotine/alcohol use disorder before | 0.99 (0.80, 1.23) | 1.20 (1.04, 1.38) | | |
| Medications for nicotine/alcohol use disorder during | 1.37 (0.90, 2.10) | 1.26 (0.93, 1.72) | | |
| Other pain medications before | 0.88 (0.71, 1.09) | 0.80 (0.67, 0.96) | | |
| Other pain medications during | 0.94 (0.65, 1.35) | 1.09 (0.80, 1.48) | | |
| SSRIs before | 1.31 (1.23, 1.39) | 1.32 (1.26, 1.39) | | |
| SSRIs during | 1.23 (1.14, 1.32) | 1.10 (1.04, 1.16) | | |
| Paracetamol before | 1.01 (0.95, 1.07) | 1.12 (1.07, 1.17) | | |
| Paracetamol during | 1.08 (1.01, 1.15) | 1.17 (1.11, 1.23) | | |
| NSAIDs before | 1.15 (1.11, 1.20) | 1.25 (1.21, 1.28) | | |
| NSAIDs during | 1.00 (0.90, 1.12) | 1.00 (0.92, 1.08) | | |
| **Birthing parent characteristics** |  |  | | |
| **Birthing parent age** |  |  | | |
| 19 or younger | 0.83 (0.75, 0.92) | 0.94 (0.88, 1.01) | | |
| 20-29 | *Reference* | *Reference* | | |
| 30-39 | 1.06 (1.03, 1.10) | 0.92 (0.89, 0.94) | | |
| 40-45 | 1.23 (1.14, 1.32) | 0.87 (0.82, 0.93) | | |
| 46 and older | 1.57 (1.15, 2.13) | 0.94 (0.67, 1.32) | | |
| **Birthing parent diagnoses before conception** | | |  |  |
| Attention-deficit/hyperactivity disorder | 1.23 (1.07, 1.42) | 1.82 (1.63, 2.02) | | |
| Autism Spectrum disorder | 2.07 (1.73, 2.49) | 1.32 (1.11, 1.56) | | |
| Definite or uncertain suicide attempt | 1.08 (1.00, 1.16) | 1.07 (1.01, 1.13) | | |
| Alcohol use disorder | 1.06 (0.97, 1.15) | 1.13 (1.07, 1.20) | | |
| Other non-tobacco substance use disorder | 0.96 (0.85, 1.08) | 1.04 (0.96, 1.13) | | |
| Serious mental illness | 1.12 (0.99, 1.26) | 1.10 (0.99, 1.22) | | |
| Non-bipolar mood disorder | 1.18 (1.11, 1.26) | 1.16 (1.11, 1.22) | | |
| Anxiety disorder | 1.25 (1.19, 1.32) | 1.15 (1.10, 1.19) | | |
| **Highest level of education** |  |  | | |
| Less than 9 years | *Reference* | *Reference* | | |
| 9 years | 1.08 (0.99, 1.19) | 1.35 (1.24, 1.46) | | |
| 1 to 3 years of upper secondary | 0.95 (0.87, 1.03) | 1.15 (1.06, 1.25) | | |
| Any post-secondary or postgraduate | 0.79 (0.73, 0.87) | 0.88 (0.81, 0.96) | | |
| **Country of origin is Sweden** | 0.73 (0.71, 0.75) | 1.67 (1.61, 1.72) | | |
| **Other familial and socioeconomic characteristics** | | |  |  |
| **Cohabitation at Childbirth** |  |  | | |
| Cohabitating | *Reference* | *Reference* | | |
| Single | 1.30 (1.22, 1.40) | 1.36 (1.29, 1.43) | | |
| Other cohabitation situation | 1.33 (1.26, 1.40) | 1.27 (1.22, 1.33) | | |
| **Birthing parent income in year before conception** | | |  |  |
| 1st quintile (lowest) | 1.27 (1.22, 1.33) | 1.06 (1.02, 1.09) | | |
| 2nd quintile | 1.10 (1.06, 1.15) | 1.04 (1.02, 1.07) | | |
| 3rd quintile | *Reference* | *Reference* | | |
| 4th quintile | 0.93 (0.89, 0.98) | 0.97 (0.94, 1.01) | | |
| 5th quintile (highest) | 0.84 (0.79, 0.90) | 0.99 (0.94, 1.03) | | |
| **Non-birthing parent age** |  |  | | |
| 19 or younger | 0.90 (0.75, 1.08) | 0.98 (0.88, 1.10) | | |
| 20-29 | *Reference* | *Reference* | | |
| 30-39 | 1.06 (1.03, 1.10) | 0.97 (0.94, 0.99) | | |
| 40-45 | 1.22 (1.15, 1.28) | 1.01 (0.97, 1.05) | | |
| 46 and older | 1.25 (1.16, 1.34) | 1.08 (1.02, 1.15) | | |
| **Non-birthing parent diagnoses before conception** |  |  | | |
| Attention-deficit/hyperactivity disorder | 1.16 (1.03, 1.32) | 1.89 (1.74, 2.05) | | |
| Autism Spectrum disorder | 1.97 (1.58, 2.46) | 1.38 (1.13, 1.68) | | |
| Definite or uncertain suicide attempt | 0.94 (0.86, 1.04) | 1.12 (1.05, 1.19) | | |
| Opioid use disorder | 1.06 (0.86, 1.31) | 0.98 (0.85, 1.14) | | |
| Alcohol use disorder | 1.15 (1.06, 1.24) | 1.22 (1.16, 1.29) | | |
| Other non-tobacco substance use disorder | 0.88 (0.79, 0.99) | 1.03 (0.96, 1.12) | | |
| Serious mental illness | 1.22 (1.05, 1.41) | 1.04 (0.93, 1.18) | | |
| Non-bipolar mood disorder | 1.21 (1.11, 1.31) | 1.10 (1.03, 1.17) | | |
| Anxiety disorder | 1.21 (1.12, 1.29) | 1.13 (1.07, 1.19) | | |
| **Non-birthing parent highest level of education** |  |  | | |
| Less than 9 years | *Reference* | *Reference* | | |
| 9 years | 1.08 (0.99, 1.18) | 1.15 (1.07, 1.24) | | |
| 1 to 3 years of upper secondary | 0.97 (0.90, 1.05) | 0.90 (0.84, 0.97) | | |
| Any post-secondary or postgraduate | 0.98 (0.90, 1.07) | 0.66 (0.62, 0.72) | | |

Abbreviations: Hazard Ratio, HR; 95% CI, 95% Confidence Interval; ADHD, attention-deficit/hyperactivity disorder;

SSRIs, selective serotonin reuptake inhibitors; NSAIDs, nonsteroidal anti-inflammatory drugs
