# Supplementary material for: Prescribed opioid analgesic use in pregnancy and risk of neurodevelopmental disorders in children: A retrospective study in Sweden
Source: PLoS Med. 2025 Sep 16;22(9):e1004721. doi: 10.1371/journal.pmed.1004721 (PMC12440195; doi:10.1371/journal.pmed.1004721)
Supplement: S1 Text — (DOCX) [file pmed.1004721.s028.docx]

**S1 Text.** Analysis plan and updates

Below is the original analysis plan. In response to reviewer comments, several changes were made which are detailed here:

Changes to original exclusion criteria:

- There were no children who needed to be dropped due to invalid identifiers
- Added exclusion due to invalid parity
- Added exclusion due to birthing parent opioid use disorder diagnosis
- Added exclusion based on non-informative prescriptions
- Added exclusion of extreme dose values
- Added exclusion for death or emigration before age 4 for ADHD analysis cohort only

Changes to covariates:

- Rather than controlling for painful conditions using covariates in the model, we used the diagnoses to restrict the cohort to only those who had a diagnosed painful condition (Model 3)

Changes to sensitivity analyses:

- We included all planned sensitivity analyses and added the following additional analyses to address potential sources of bias:
  - Excluding “as needed” prescriptions
  - Using number of prescriptions rather than dose and duration derived from text-mining
  - Using continuous dose and duration of exposure
  - Examining sensitive periods (early vs middle/late pregnancy) of exposure for dose and duration of exposure
  - Using covariates measured before conception only
  - Using outcomes based on first/earliest diagnosis of ASD or ADHD after birth (rather than after age 2,4)

-----------------------------------------------------------------------------------------

Created: 2023-03-07 by Emma Cleary

Updated:2024-02-27 by Emma Cleary

# OBJECTIVE AND HYPOTHESES

The aim of this study is to assess the risk of two offspring neurodevelopmental disorders, attention-deficit/hyperactivity disorder (ADHD) and autism spectrum disorder (ASD) as a function of cumulative dose and duration of prescribed opioid analgesic exposure during pregnancy. The hypothesis of this study is that exposure to higher doses and longer duration of treatment will be associated with increased risks of ASD and ADHD, which will be attenuated after adjustment for confounding.

# NOTATION AND ABBREVATIONS

*XXX explanation*

POA Prescribed Opioid Analgesic

ADHD Attention-deficit/hyperactivity disorder

ASD Autism Spectrum Disorder

ICD International Classification of Diseases

ATC Anatomical Therapeutic Chemical

# STUDY POPULATION

## INCLUSION CRITERIA

Starting cohort of 1,287,495 births in Sweden restricted to births between July 1^st^, 2007 and December 31^st^ 2018 with follow-up until December 31^st^, 2021. To allow for a minimum of 6 years of follow-up for ADHD, those models only include births through December 31^st^ 2015.

## EXCLUSION CRITERIA

Children with invalid identifiers

Children born to birthing parent with invalid identifiers

Children with invalid sex

Children born to birthing parents who filled a prescription for Buprenorphine or Methadone or Naltrexone within 1 year before conception or during pregnancy

Stillbirths or neonatal deaths within 27 days after birth

Births that died or emigrated before age 2

# MEASUREMENTS AND VARIABLES

## Outcome

First ASD diagnosis after age 2 (identified through ICD Codes F84, eventually excluding F84.2)

First ADHD diagnosis after age 4 (ICD Codes F90)

ADHD cases also identified through offspring ADHD medication use (first dispensation of ATC codes: N06BA01, N06BA02, N06BA04, N06BA09, N06BA12)

## Exposure covariates

The primary exposure will be cumulative opioid dose during pregnancy (converted to oral morphine milligram equivalents). The exposure will be identified through birthing parent N02A (ATC code) dispensations binned in order to compare outcomes associated with higher and lower doses. Dispensations in the day before or day of birth were not included to avoid capturing labor or post-delivery analgesia.

Secondary exposure definitions that will be examined will include cumulative # of days of exposure during pregnancy (based on predicted maximum dose). The exposure will be binned in order to compare outcomes associated with shorter and longer durations.

Pregnancy covariates:

Year of birth

Parity

Multiple births

Smoking 3 months before pregnancy

Smoking during 1^st^ trimester

Exposure to other pain and psychoactive medications (birthing parent prescriptions) during pregnancy (concomitant medication use) and within 1 year before conception

## Mandatory covariates, known confounders

Birthing parent and partner age at conception

Birthing parent and partner highest level of education at year before conception

Birthing parent and partner psychiatric diagnoses before conception

Parental cohabitation status

Birthing parent income at year before conception

Birthing parent country of origin

****Reasons for including these covariates*****

Previous research on associations between mental health, family background, and opioid analgesic use in Sweden provided guidance on background characteristics that may confound associations between POA use during pregnancy and offspring ADHD and ASD.

- Sujan, A. C. et al. Maternal prescribed opioid analgesic use during pregnancy and associations with adverse birth outcomes: A population-based study. PLoS Med 16, e1002980, doi:10.1371/journal.pmed.1002980 (2019)
- Sujan, A. C. et al. A nation-wide Swedish study of opioid analgesic prescribing patterns during pregnancy and associated preexisting mental health conditions. J Matern Fetal Neonatal Med, 1- 7, doi:10.1080/14767058.2021.1875436 (2021)
- Quinn, P. D. et al. Associations of mental health and family background with opioid analgesic therapy: a nationwide Swedish register-based study. Pain 160, 2464-2472, doi:10.1097/j.pain.0000000000001643 (2019).]

## Additional covariates, potential confounders

Birthing parent painful conditions to control for cofounding by indication (see table below- work in progress). Groupings of ICD codes/types of pain will be included as 9 separate covariates:

Musculoskeletal Disorders

Orofacial and headache disorders

Disease of the urinary and genital organs

Pregnancy-related

Functional disorders of the intestine

Other pain-inducing conditions (excl. cancer)

Cancer and tumors

Not otherwise classified pain-defined diagnoses

Acute conditions

****Reasons for including these covariates*****

The underlying indication for treatment with prescribed opioid analgesics (painful conditions) may also be a cause of the outcomes.

- Tronnes, J. N. et al. Association of Timing and Duration of Prenatal Analgesic Opioid Exposure With Attention-Deficit/Hyperactivity Disorder in Children. JAMA Netw Open 4, e2124324, doi:10.1001/jamanetworkopen.2021.24324 (2021).

## Effect Modification

Effect modification will not be explored in the current study

# DATA MANAGEMENT

*** raw data files, program files etc. and documentation files ***

The files in this project are stored in P:\xxxx.

The logbook (LOGBOOK_ DDP_POA_NDD.doc) can be found in Documents folder.

# STATISTICAL ANALYSES

In order to understand absolute risk of ASD and ADHD in our sample by a given age, we will use Kaplan-Meier estimation, stratified by different levels of POA exposure for dose and duration.

Next, we will run several Cox proportional hazards regression models with the primary and secondary exposure definitions (with no exposure as the reference, but will make comparisons between the HRs for the various exposure conditions) while adjusting for different sources of confounding

1. Model 1 will estimate unadjusted associations. This model will also account for clustering of individuals within nuclear families (siblings).
2. Model 2 will add statistical adjustment with measured covariates.
3. Modle 3 will estimate risks in a subset of the cohort with diagnosed painful conditions while adjusting for measured covariates.
4. Model 4 will estimate risks with a comparison group of those who received POAs before pregnancy while adjusting for measured covariates.
5. Model 5 will use a sibling comparison design to control for unmeasured genetic and environmental factors that are stable across differentially exposed siblings while also statistically adjusting for measured covariates.

We will then do several sensitivity analyses to test the assumptions of the above models as well as explore different exposure definitions.

These analyses will include:

- Re-running analyses based on the predicted minimum dosage
- Re-running analyses with exposure variables that did not include “bumping” of overlapping POA dispensation intervals
- Re-running analyses with exposure variables based only on POA dispensations in the first two trimesters

# STAFF LIST

Emma Cleary, PhD student, first author, Ayesha Sujan, co-author, Patrick Quinn, co-author, Martin Rickert, data manager, co-author

Franziska Fischer, co-author, A.Sara Oberg, co-author, Paul Lichtenstein, co-author, Brian D’Onofrio, PI, senior author
