# Supplementary material for: Prescribed opioid analgesic use in pregnancy and risk of neurodevelopmental disorders in children: A retrospective study in Sweden
Source: PLoS Med. 2025 Sep 16;22(9):e1004721. doi: 10.1371/journal.pmed.1004721 (PMC12440195; doi:10.1371/journal.pmed.1004721)
Supplement: S2 Text — (DOCX) [file pmed.1004721.s029.docx]

**S2 Text.** Text-mining and exposure definitions

Our approach to predicting dosage from free-text prescription data follows directly from, and expands upon, our previous work [Zhang L*, Lagerberg T*, Chen Q, et al. Prediction of treatment dosage and duration from free-text prescriptions: an application to ADHD medications in the Swedish prescribed drug register. *Evid Based Ment Health.* 2021;24(4):146-152] Opioids have more varied and complex prescriptions than ADHD medications, thus we developed a range of models to capture many features of opioid prescriptions.

First, we used algorithms and components implemented in Python 3.7 available in the KerasNLP natural language processing library. Five separate classifier models (NLP1-NLP5) were trained on a sample of 8000 prescriptions for medications administered in discrete form in the years 2006, 2010, and 2012. The proportion of ATCs for the most frequent prescription texts in the training data were selected to correspond to the ATC proportions as represented in the total Prescription Drug Register (PDR) during 2006 + 2010 + 2012. The five NLP models were:

- NLP1 predicted a binary variable indicating whether the prescription text was informative (i.e., dosage information could be determined)
- NLP2 predicted a binary variable indicating whether the prescription was to be taken on an “as-needed” basis (versus “scheduled”)
- NLP3 predicted a binary variable indicating whether the free-text specified a range of acceptable dosages (i.e., daily min and max differed)
- NLP4 predicted an ordinal categorical variable representing the daily minimum dosage (“0.5”, “1”, “2”, …, “9+”)
- NLP5 predicted an ordinal categorical variable representing the daily maximum dosage (“0.5”, “1”, “2”, …, “9+”)

Pre-processing of the training data was performed in Python. Specifically, we removed unnecessary punctuation (e.g., “.”, “!” etc.), grammatical inflections (e.g., “dagen” replaced with “dag”), and common Swedish stopwords (e.g., “en”, “till”) listed in the Natural Language Toolkit (NLTK 3.2.5) corpus. Unique words in the text were tokenized (i.e., coded as numbers), and each sequence of tokens was padded with zeros to ensure the inputs to the model were the same length. The pre-processed data were then inputted to the models, each one consisting of four sequential layers:

- An embedding layer (words are represented as in vector space so that computations can be made on their inter-relationships, and so that words appearing close to each other in vector space are more similar in meaning. Classic example of how combinations of words can be used to surmise meaning in this set-up: “king”-“man”+”woman”=”queen”)
- A flattening layer (making input into a one-dimensional vector for input in the following layers)
- A dense hidden layer (8 nodes for model NLP1-3; 64 nodes for model NLP4-5) with a rectified linear (ReLU) activation function
- A dense output layer (1 node in model NLP1-3 as there is only one binary (0,1) output; 10 nodes in model NLP4-5 as there are 10 outputs in the form of the different categories of daily dosage

We performed both internal and external validation of the five natural language processing models that were trained separately to predict three binary variables (whether the prescription text is informative, whether a range of dosages is specified, and if the dose should be taken on an as-needed basis), as well as the minimum and maximum daily dosage from free-text prescription information available in the PDR.  We assessed model performance using simple accuracy and tetra/polychoric correlations as validation statistics.

For purposes of internal validation, each model was trained on a sample of 8000 of the most frequently prescribed opioids administered in discrete units (i.e., pills) in 2006, 2010, and 2012. The proportion of an ATC code in the training sample was adjusted to approximately match the proportion in the PDR register in those years.  We found that the internal validation accuracy was excellent for each of the predicted variables, i.e., 99.86% for non-informative, 99.30% for range, 94.13% for as needed, 98.63% for minimum number of pills, and 91.69% for maximum number of pills.

For purposes of external validation, two independent researchers extracted the gold standard dosage for random sample of 1000 prescription texts from the text-mining prediction data, with a quality check by a third researcher.  The estimated tetra/polychoric correlations between the model predictions and the gold standard reading were 0.99 for non-informative, 0.98 for range, 0.94 for as needed, 0.96 for minimum number of pills, and 0.95 for maximum number of pills.

*OME conversion and missing information*

Nielsen table was used for OME conversion [Nielsen, S., Degenhardt, L., Hoban, B., & Gisev, N. (2016). A synthesis of oral morphine equivalents (OME) for opioid utilisation studies. *Pharmacoepidemiology and drug safety*, *25*(6), 733-737.]

Some formulations were missing information on conversion factors, we used the following for OME conversion:

| **Substance** | **Preparation** | **Available concentrations** |
| --- | --- | --- |
| Ketobemidon | ospecificerad | *Preparation form not given* |
| Morfin | oral lösning | 2 to 20 mg/ml |
| Morfin | injektionsvätska | 1 to 20 mg/ml |
| Morfin | ospecificerad | *Preparation form not given* |
| Oxikodon | oral lösning | 1 to 10 mg/ml |
| Tramadol | orala droppar | 100 mg/ml* |
| Tramadol | oral lösning | *Preparation form not found -* *assume same as for oral drops above* |
| Tramadol | injektionsvätska | 50 mg/ml |

*Matching on non-informative prescriptions:*

14,075 prescriptions were deemed to be “non-informative,” meaning that the algorithm was not able to predict a daily minimum and maximum value. The majority of these prescriptions were to individuals who also had at least one informative prescription. We therefore imputed the information for these non-informative prescriptions by matching them with other, similar informative prescriptions. Matching prescriptions were those with the same ATC code, the same formulation, from the same type of clinic, and the same strength. If there were multiple prescriptions that matched these criteria, we selected the one that was closest in time to the non-informative prescription. Pregnancies with only non-informative prescriptions (S1 Table) were excluded.

*Identifying treatment intervals and shifting similar prescriptions:*

We calculated the expected treatment interval length/duration (in days) based on the selected dosage (either daily minimum or daily maximum) per day. We used the following algebraic expression for this calculation:

$Expected treatment length \left( days \right)= \frac{\left( Number of pills per package \times Package size \right)}{Prescribed daily dosage}$

Overlapping prescriptions with the same ATC, strength, and formulation (both immediate or both extended release) were assumed to have not been taken concurrently and the start date of the later prescription was shifted so that the start date was the next date after the final day of the first prescription, to account for possible medication “stockpiling”. Thus, compared with the initial partially overlapping intervals, the concatenated interval duration was increased by the number of days of overlap (see Δ S1 Fig). This could have inflated the number of exposed pregnancies as well as the amount of exposure during the pregnancy interval. In case our assumption that these prescriptions were not being taken at the same time was incorrect, we re-ran analyses with dose and duration variables that did not include this shift in a sensitivity analysis (see S2 Fig).
