# Supplementary material for: Prescribed opioid analgesic use in pregnancy and risk of neurodevelopmental disorders in children: A retrospective study in Sweden
Source: PLoS Med. 2025 Sep 16;22(9):e1004721. doi: 10.1371/journal.pmed.1004721 (PMC12440195; doi:10.1371/journal.pmed.1004721)
Supplement: S3 Text — (DOCX) [file pmed.1004721.s030.docx]

**S3 Text.** Trimester definitions

*Identifying prescriptions by trimester and that overlap with pregnancy interval*

We first estimated last menstrual period (LMP) by subtracting gestational age (predominantly based on ultrasound measurements occurring the 18th to 20th week of pregnancy) from birth date and then estimated conception date by adding 14 days to LMP.

For the sensitivity analyses of sensitive periods of exposure (see S17 Table & S18 Table) the following trimester definitions were used:

· First trimester: conception date to 76 days after conception

· Second trimester: 77 to 166 days after conception

· Third trimester: 167 days after conception through day before birth
