# Supplementary material for: Prescribed opioid analgesic use in pregnancy and risk of neurodevelopmental disorders in children: A retrospective study in Sweden
Source: PLoS Med. 2025 Sep 16;22(9):e1004721. doi: 10.1371/journal.pmed.1004721 (PMC12440195; doi:10.1371/journal.pmed.1004721)
Supplement: S4 Text — (DOCX) [file pmed.1004721.s031.docx]

**S4 Text**. SAS 9.4 (TS Level 1M6) code for primary analyses and continuous sensitivity analyses.

All analyses listed below were run on a remote Red Hat Enterprise Linux 7.9 compute server with 56 CPU threads and 768 GB of RAM.

***

*** [0] LIBRARY REFS

***;

libname scratch '/scratch/tmp/emmcle'; * Pointer to scratch disk (500GB);

libname data 'projects/DDP_POA/ndd_data'; * Pointer to permanent files in personal project directory;

libname src 'projects/DDP_POA/ndd_source'; * Pointer to original source files in personal project directory;

libname res 'projects/DDP_POA/ndd_results'; * Pointer to new place for permanent created files in personal project directory;

***

*** [1] UNADJUSTED MODELS (with robust sandwich variance estimator)

***;

/* Models A-D use the categorical exposure as the predictor*/

/* Model A, ome_median_max exposure, population wide, ASD */

**proc** **phreg** data=data.cohort_nomiss covs(aggregate);

where Primary_cohort_ASD=**1**;

class ome_median_max (ref='0');

model age_asd*asd(**0**) = ome_median_max / risklimits=wald;

id PINm;

ods output ParameterEstimates=scratch.parms_A;

**run**;

/* Model B, duration_weeks_max exposure, population wide, ASD */

**proc** **phreg** data=data.cohort_nomiss covs(aggregate);

where Primary_cohort_ASD=**1**;

class duration_weeks_max (ref='0');

model age_asd*asd(**0**) = duration_weeks_max / risklimits=wald;

id PINm;

ods output ParameterEstimates=scratch.parms_B ;

**run**;

/* Model C, ome_median_max exposure, population wide, ADHD */

**proc** **phreg** data=data.cohort_nomiss covs(aggregate);

where Primary_cohort_ADHD=**1** ;

class ome_median_max (ref='0');

model age_adhd_dxrx*adhd_dxrx(**0**) = ome_median_max / risklimits=wald;

id PINm;

ods output ParameterEstimates=scratch.parms_C ;

**run**;

/* Model D, duration_weeks_max exposure, population wide, ADHD */

**proc** **phreg** data=data.cohort_nomiss covs(aggregate);

where Primary_cohort_ADHD=**1**;

class duration_weeks_max (ref='0');

model age_adhd_dxrx*adhd_dxrx(**0**) = duration_weeks_max / risklimits=wald;

id PINm;

ods output ParameterEstimates=scratch.parms_D ;

**run**;

/* Models E-H use the original, continuous exposure as the predictor */

/*Model E, ome_median_max exposure, population wide, ASD*/

**proc** **phreg** data=data.cohort_nomiss covs(aggregate);

where Primary_cohort_ASD=**1**;

model age_asd*asd(**0**) = ome_cont / risklimits=wald; /*scaled per 100mg*/

id PINm;

ods output ParameterEstimates=scratch.parms_E ;

**run**;

/*Model F, duration_weeks_max exposure, population wide, ASD*/

**proc** **phreg** data=data.cohort_nomiss covs(aggregate);

where Primary_cohort_ASD=**1**;

model age_asd*asd(**0**) = duration_cont / risklimits=wald; /*scaled per 7 days*/

id PINm;

ods output ParameterEstimates=scratch.parms_F ;

**run**;

/*Model G, ome_median_max exposure, population wide, ADHD*/

**proc** **phreg** data=data.cohort_nomiss covs(aggregate);

where Primary_cohort_ADHD=**1** ;

model age_adhd_dxrx*adhd_dxrx(**0**) = ome_cont / risklimits=wald;

id PINm;

ods output ParameterEstimates=scratch.parms_G ;

**run**;

/*Model H, duration_weeks_max exposure, population wide, ADHD*/

**proc** **phreg** data=data.cohort_nomiss covs(aggregate);

where Primary_cohort_ADHD=**1**;

model age_adhd_dxrx*adhd_dxrx(**0**) = duration_cont / risklimits=wald;

id PINm;

ods output ParameterEstimates=scratch.parms_H ;

**run**;

***

*** [2] COVARIATE ADJUSTED MODELS

***;

/* Notes:

(1) define a macro variable ‘covs_adj’ with the covariates that will be included in all adjusted models except _bp SA,

(2) reference level commented out for categorical variables

(3) _wm is ‘with missing’ representing that before multiple imputation, all non-birthing parent variables had some missing (see S11 table)

*/

%let covs_adj =

/* Categorical covariates */

/*ROK0_cat: Maternal smoking before pregnancy */

/*smoke_b_none*/ smoke_b_1to9 smoke_b_10plus

/*ROK1_cat: Maternal smoking */

/*smoke_d_none*/ smoke_d_1to9 smoke_d_10plus

/*cohab_cat: Cohabitation status */

/*cohab_together*/ cohab_single cohab_other

/*PAC_cat: Paternal age (y) at childbirth */

PAC_le19_mi /*PAC_20_29_mi*/ PAC_30_39_mi PAC_40_45_mi PAC_45p_mi

/*educ_m_cat: Maternal highest education level attained */

/*m_edu_less9_mi*/ m_edu_9_mi m_edu_1to3up_mi m_edu_post_mi

/*educ_f_cat: Paternal highest education level attained */

/*f_edu_less9_mi*/ f_edu_9_mi f_edu_1to3up_mi f_edu_post_mi

/*MAC_cat: Maternal age (y) at childbirth */

MAC_le19_mi /*MAC_20_29_mi*/ MAC_30_39_mi MAC_40_45_mi MAC_45p_mi

/*parity_cat: Birth parity*/

/*parity1_mi*/ parity2_mi parity3_mi parity4p_mi

/*yearb_cat: Child’s year of birth*/

/*yearb_07_10_mi*/ yearb_11_14_mi yearb_15_18_mi

/*minc_cat: Maternal income (quintile)*/

minc_1Q minc_2Q /*minc_3Q*/ minc_4Q minc_5Q

/*Binary covariates*/

born_sweden_m

female_recode

multiple_birth

dx_opioid_f1_wm

dx_alcohol_m1

dx_alcohol_f1_wm

dx_nontobac_m1

dx_nontobac_f1_wm /*nontobacco SUD*/

dx_smi_m1

dx_smi_f1_wm

dx_nonbipmood_m1

dx_nonbipmood_f1_wm

dx_anx_m1

dx_anx_f1_wm

dx_sui_m1

dx_sui_f1_wm

/*parental asd and adhd diagnoses (dx) */

dx_asd_m1 /*mother*/

dx_asd_f1_wm /*father*/

dx_adhd_m1

dx_adhd_f1_wm

/*maternal meds before & during */

adhd_m_b /*for 1 yr before conception*/

adhd_m_d /*during pregnancy*/

anticonv_m_d

anticonv_m_b

anticonv_lith_m_d

anticonv_lith_m_b

antipsy_m_d

antipsy_m_b

anxio_m_d

anxio_m_b

benzo_m_d

benzo_m_b

benzo_z_m_d

benzo_z_m_b

cycl_m_d

cycl_m_b

hypsed_m_d

hypsed_m_b

migr_m_d

migr_m_b

nico_alc_m_d

nico_alc_m_b

other_pain_m_d

other_pain_m_b

ssri_m_d

ssri_m_b

paracet_m_b

paracet_m_d

nsaidsasp_m_b

nsaidsasp_m_d;

/* Analysis sample for covariate-adjusted ASD models (categorical predictor, then continuous)*/

**data** scratch.Primary_cohort_ASD;

set data.cohort_nomiss (where=(Primary_cohort_ASD=**1**));

**run**;

/* Macro for population-wide categorical ASD Models 1-6 (with robust sandwich variance estimator) */

**%macro** mCox_asd(_DATAFL=,_EXPOSURE=, _MDL= );

proc phreg data=scratch.Primary_cohort_ASD %if %bquote(&_DATAFL.) ne %then %str((where=(&_DATAFL.))); covs(aggregate);

class &_EXPOSURE. (ref="0");

id PINm;

by _Imputation_;

model age_asd * asd(**0**) = &_EXPOSURE. &covs_adj / risklimits = wald;

ods output ParameterEstimates=scratch.parms_&_MDL.;

proc mianalyze parms(CLASSVAR=CLASSVAL)=scratch.parms_&_MDL.;

CLASS &_EXPOSURE.;

modeleffects &_EXPOSURE. &covs_adj;

ods output ParameterEstimates=scratch.parms_&_MDL._exp;

data scratch.HR_asd_cat_&_MDL.;

set scratch.parms_&_MDL._exp;

HR = exp(estimate);

LCL = exp(LCLMean);

UCL = exp(UCLMean);

run;

**%mend**;

/*Model 1, ome_median_max exposure, population wide, ASD, cov adjustment*/

%***mCox_asd***( _DATAFL =

, _EXPOSURE = ome_median_max

, _MDL = **1**

);

/*Model 2, duration_weeks_max exposure, population wide, ASD, cov adjustment*/

%***mCox_asd***( _DATAFL =

, _EXPOSURE = duration_weeks_max

, _MDL = **2**

);

/*Model 3, ome_median_max exposure, any_pain, ASD, cov adjustment*/

%***mCox_asd***( _DATAFL = any_pain=**1**

, _EXPOSURE = ome_median_max

, _MDL = **3**

);

/*Model 4, duration_weeks_max exposure, any_pain, ASD, cov adjustment*/

%***mCox_asd***( _DATAFL = any_pain=**1**

, _EXPOSURE = duration_weeks_max

, _MDL = **4**

);

/*Model 5, ome_median_max exposure, either_b_d, ASD, cov adjustment*/

%***mCox_asd***( _DATAFL = either_b_d=**1**

, _EXPOSURE = ome_median_max

, _MDL = **5**

);

/*Model 6, duration_weeks_max exposure, either_b_d, ASD, cov adjustment*/

%***mCox_asd***( _DATAFL = either_b_d=**1**

, _EXPOSURE = duration_weeks_max

, _MDL = **6**

);

/*Now define the macro for the covariate-adjusted sibling comparisons categorical ASD Models 7 & 8 */

**%macro** mCox_asd_sib(_DATAFL=,_EXPOSURE=, _MDL= );

proc phreg data=scratch.Primary_cohort_ASD %if %bquote(&_DATAFL.) ne %then %str((where=(&_DATAFL.))); NOSUMMARY;

class &_EXPOSURE. (ref="0");

strata PINm;

by _Imputation_;

model age_asd * asd(**0**) = &_EXPOSURE. &covs_adj / risklimits = wald;

ods output ParameterEstimates=scratch.parms_&_MDL.;

proc mianalyze parms(CLASSVAR=CLASSVAL)=scratch.parms_&_MDL.;

CLASS &_EXPOSURE.;

modeleffects &_EXPOSURE.; /*skip printing of covariates*/

ods output ParameterEstimates=scratch.parms_&_MDL._exp;

data scratch.HR_asd_cat_sib_&_MDL.;

set scratch.parms_&_MDL._exp;

HR = exp(estimate);

LCL = exp(LCLMean);

UCL = exp(UCLMean);

run;

**%mend**;

/*Model 7, ome_median_max exposure, sib, ASD, cov adjustment*/

%***mCox_asd_sib***( _DATAFL =

, _EXPOSURE = ome_median_max

, _MDL = **7**

);

/*Model 8, duration_weeks_max exposure, sib, ASD, cov adjustment*/

%***mCox_asd_sib***( _DATAFL =

, _EXPOSURE = duration_weeks_max

, _MDL = **8**

);

/*Next, macro for population wide continuous ASD Models 1-6 (with robust sandwich variance estimator)*/

**%macro** mCox_asd_con(_DATAFL=,_EXPOSURE=, _MDL= );

proc phreg data=scratch.Primary_cohort_ASD %if %bquote(&_DATAFL.) ne %then %str((where=(&_DATAFL.))); covs(aggregate);

id PINm;

by _Imputation_;

model age_asd * asd(**0**) = &_EXPOSURE. &covs_adj / risklimits = wald;

ods output ParameterEstimates=scratch.parms_&_MDL.;

proc mianalyze parms /*(CLASSVAR=CLASSVAL)*/=scratch.parms_&_MDL.;

modeleffects &_EXPOSURE.;

ods output ParameterEstimates=scratch.parms_&_MDL._exp;

data scratch.HR_asd_con_&_MDL.;

set scratch.parms_&_MDL._exp;

HR = exp(estimate);

LCL = exp(LCLMean);

UCL = exp(UCLMean);

run;

**%mend**;

/*Model 1, ome_median_max exposure, population wide, ASD, cov adjustment*/

%***mCox_asd_con***( _DATAFL =

, _EXPOSURE = ome_cont

, _MDL = **1**

);

/*Model 2, duration_weeks_max exposure, population wide, ASD, cov adjustment*/

%***mCox_asd_con***( _DATAFL =

, _EXPOSURE = duration_cont

, _MDL = **2**

);

/*Model 3, ome_median_max exposure, any_pain, ASD, cov adjustment*/

%***mCox_asd_con***( _DATAFL = any_pain=**1**

, _EXPOSURE = ome_cont

, _MDL = **3**

);

/*Model 4, duration_weeks_max exposure, any_pain, ASD, cov adjustment*/

%***mCox_asd_con***( _DATAFL = any_pain=**1**

, _EXPOSURE = duration_cont

, _MDL = **4**

);

/*Model 5, ome_median_max exposure, either_b_d, ASD, cov adjustment*/

%***mCox_asd_con***( _DATAFL = either_b_d=**1**

, _EXPOSURE = ome_cont

, _MDL = **5**

);

/*Model 6, duration_weeks_max exposure, either_b_d, ASD, cov adjustment*/

%***mCox_asd_con***( _DATAFL = either_b_d=**1**

, _EXPOSURE = duration_cont

, _MDL = **6**

);

/*next, define macro for continuous ASD sib Models 7 & 8*/

**%macro** mCox_asd_con_sib(_DATAFL=, _EXPOSURE=, _MDL= );

proc phreg data=scratch.Primary_cohort_ASD %if %bquote(&_DATAFL.) ne %then %str((where=(&_DATAFL.))); NOSUMMARY;

strata PINm;

by _Imputation_;

model age_asd * asd(**0**) = &_EXPOSURE. &covs_adj / risklimits = wald;

ods output ParameterEstimates=scratch.parms_&_MDL.;

proc mianalyze parms /*(CLASSVAR=CLASSVAL)*/=scratch.parms_&_MDL.;

modeleffects &_EXPOSURE.;

ods output ParameterEstimates=scratch.parms_&_MDL._exp;

data scratch.HR_asd_con_sib_&_MDL.;

set scratch.parms_&_MDL._exp;

HR = exp(estimate);

LCL = exp(LCLMean);

UCL = exp(UCLMean);

run;

**%mend**;

/*Model 7, ome_median_max exposure, sib , ASD, cov adjustment*/

%***mCox_asd_con_sib***( _DATAFL =

, _EXPOSURE = ome_cont

, _MDL = **7**

);

/*Model 8, duration_weeks_max exposure,sib, ASD, cov adjustment*/

%***mCox_asd_con_sib***( _DATAFL =

, _EXPOSURE = duration_cont

, _MDL = **8**

);

/* Analysis sample for covariate-adjusted ADHD models (categorical, then continuous)*/

**data** scratch.Primary_cohort_ADHD;

set data.cohort_nomiss (where=(Primary_cohort_ADHD=**1**));

**run**;

/*Macro for population-wide categorical ADHD Models 1-6 (with robust sandwich variance estimator)*/

**%macro** mCox_adhd(_DATAFL=,_EXPOSURE=, _MDL= );

proc phreg data=scratch.Primary_cohort_ADHD %if %bquote(&_DATAFL.) ne %then %str((where=(&_DATAFL.))); covs(aggregate);

class &_EXPOSURE. (ref="0");

id PINm;

by _Imputation_;

model age_adhd_dxrx*adhd_dxrx(**0**) = &_EXPOSURE. &covs_adj / risklimits = wald;

ods output ParameterEstimates=scratch.parms_&_MDL.;

proc mianalyze parms(CLASSVAR=CLASSVAL)=scratch.parms_&_MDL.;

CLASS &_EXPOSURE.;

modeleffects &_EXPOSURE. &covs_adj;

ods output ParameterEstimates=scratch.parms_&_MDL._exp;

data scratch.HR_adhd_cat_&_MDL.;

set scratch.parms_&_MDL._exp;

HR = exp(estimate);

LCL = exp(LCLMean);

UCL = exp(UCLMean);

run;

**%mend**;

/*Model 1, ome_median_max exposure, population wide, ADHD, cov adjustment*/

%***mCox_adhd***( _DATAFL =

, _EXPOSURE = ome_median_max

, _MDL = **1**

);

/*Model 2, duration_weeks_max exposure, population wide, ADHD, cov adjustment*/

%***mCox_adhd***( _DATAFL =

, _EXPOSURE = duration_weeks_max

, _MDL = **2**

);

/*Model 3, ome_median_max exposure, any_pain, ADHD, cov adjustment*/

%***mCox_adhd***( _DATAFL = any_pain=**1**

, _EXPOSURE = ome_median_max

, _MDL = **3**

);

/*Model 4, duration_weeks_max exposure, any_pain, ADHD, cov adjustment*/

%***mCox_adhd***( _DATAFL = any_pain=**1**

, _EXPOSURE = duration_weeks_max

, _MDL = **4**

);

/*Model 5, ome_median_max exposure, either_b_d, ADHD, cov adjustment*/

%***mCox_adhd***( _DATAFL = either_b_d=**1**

, _EXPOSURE = ome_median_max

, _MDL = **5**

);

/*Model 6, duration_weeks_max exposure, either_b_d, ADHD, cov adjustment*/

%***mCox_adhd***( _DATAFL = either_b_d=**1**

, _EXPOSURE = duration_weeks_max

, _MDL = **6**

);

/*Define macro for categorical sib ADHD Models 7 & 8 */

**%macro** mCox_adhd_sib(_DATAFL=,_EXPOSURE=, _MDL= );

proc phreg data=scratch.Primary_cohort_ADHD %if %bquote(&_DATAFL.) ne %then %str((where=(&_DATAFL.))); NOSUMMARY;

class &_EXPOSURE. (ref="0");

strata PINm;

by _Imputation_;

model age_adhd_dxrx*adhd_dxrx(**0**) = &_EXPOSURE. &covs_adj / risklimits = wald;

ods output ParameterEstimates=scratch.parms_&_MDL.;

proc mianalyze parms(CLASSVAR=CLASSVAL)=scratch.parms_&_MDL.;

CLASS &_EXPOSURE.;

modeleffects &_EXPOSURE.;

ods output ParameterEstimates=scratch.parms_&_MDL._exp;

data scratch.HR_adhd_cat_sib_&_MDL.;

set scratch.parms_&_MDL._exp;

HR = exp(estimate);

LCL = exp(LCLMean);

UCL = exp(UCLMean);

run;

**%mend**;

/*Model 7, ome_median_max exposure, population wide, ADHD, cov adjustment*/

%***mCox_adhd_sib***( _DATAFL =

, _EXPOSURE = ome_median_max

, _MDL = **7**

);

/*Model 8, duration_weeks_max exposure, population wide, ADHD, cov adjustment*/

%***mCox_adhd_sib***( _DATAFL =

, _EXPOSURE = duration_weeks_max

, _MDL = **8**

);

/*Macro for continuous ADHD Models 1-6 (with robust sandwich variance estimator)*/

**%macro** mCox_adhd_con(_DATAFL=,_EXPOSURE=, _MDL= );

proc phreg data=scratch.Primary_cohort_ADHD %if %bquote(&_DATAFL.) ne %then %str((where=(&_DATAFL.))); covs(aggregate);

id PINm;

by _Imputation_;

model age_adhd_dxrx*adhd_dxrx(**0**) = &_EXPOSURE. &covs_adj / risklimits = wald;

ods output ParameterEstimates=scratch.parms_&_MDL.;

proc mianalyze parms /*(CLASSVAR=CLASSVAL)*/=scratch.parms_&_MDL.;

modeleffects &_EXPOSURE.;

ods output ParameterEstimates=scratch.parms_&_MDL._exp;

data scratch.HR_adhd_con_&_MDL.;

set scratch.parms_&_MDL._exp;

HR = exp(estimate);

LCL = exp(LCLMean);

UCL = exp(UCLMean);

run;

**%mend**;

/*Model 1, ome_median_max exposure, population wide, ADHD, cov adjustment*/

%***mCox_adhd_con***( _DATAFL =

, _EXPOSURE = ome_cont

, _MDL = **1**

);

/*Model 2, duration_weeks_max exposure, population wide, ADHD, cov adjustment*/

%***mCox_adhd_con***( _DATAFL =

, _EXPOSURE = duration_cont

, _MDL = **2**

);

/*Model 3, ome_median_max exposure, any_pain, ADHD, cov adjustment*/

%***mCox_adhd_con***( _DATAFL = any_pain=**1**

, _EXPOSURE = ome_cont

, _MDL = **3**

);

/*Model 4, duration_weeks_max exposure, any_pain, ADHD, cov adjustment*/

%***mCox_adhd_con***( _DATAFL = any_pain=**1**

, _EXPOSURE = duration_cont

, _MDL = **4**

);

/*Model 5, ome_median_max exposure, either_b_d, ADHD, cov adjustment*/

%***mCox_adhd_con***( _DATAFL = either_b_d=**1**

, _EXPOSURE = ome_cont

, _MDL = **5**

);

/*Model 6, duration_weeks_max exposure, either_b_d, ADHD, cov adjustment*/

%***mCox_adhd_con***( _DATAFL = either_b_d=**1**

, _EXPOSURE = duration_cont

, _MDL = **6**

);

/*Macro for ADHD continuous sib Models 7 & 8*/

**%macro** mCox_adhd_con_sib(_DATAFL=,_EXPOSURE=, _MDL= );

proc phreg data=scratch.Primary_cohort_ADHD %if %bquote(&_DATAFL.) ne %then %str((where=(&_DATAFL.))); NOSUMMARY;

strata PINm;

by _Imputation_;

model age_adhd_dxrx*adhd_dxrx(**0**) = &_EXPOSURE. &covs_adj / risklimits = wald;

ods output ParameterEstimates=scratch.parms_&_MDL.;

proc mianalyze parms/*(CLASSVAR=CLASSVAL)*/=scratch.parms_&_MDL.;

modeleffects &_EXPOSURE.;

ods output ParameterEstimates=scratch.parms_&_MDL._exp;

data scratch.HR_adhd_con_sib_&_MDL.;

set scratch.parms_&_MDL._exp;

HR = exp(estimate);

LCL = exp(LCLMean);

UCL = exp(UCLMean);

run;

**%mend**;

/*Model 7, ome_median_max exposure, population wide, ADHD, cov adjustment*/

%***mCox_adhd_con_sib***( _DATAFL =

, _EXPOSURE = ome_cont

, _MDL = **7**

);

/*Model 8, duration_weeks_max exposure, population wide, ADHD, cov adjustment*/

%***mCox_adhd_con_sib***( _DATAFL =

, _EXPOSURE = duration_cont

, _MDL = **8**

);
